# Heterotic potential, combining ability, and stability analysis for yield and quality attributes in eggplant (*Solanum melanogena*)

**DOI:** 10.3389/fpls.2026.1836360

**Published:** 2026-06-30

**Authors:** Daleep Kumar, R. K. Narolia, Sandeep Indurthi, Tarun Kumar Meena, Atma Ram Meena, Pawan Kumar, Jitendra Gurjar, Bhupender, Hardat Kaswan

**Affiliations:** 1 Swami Keshwanand Rajasthan Agricultural University (SKRAU), Bikaner, Rajasthan, India; 2 Sri Karan Narendra Agriculture University (SKNAU), Jobner, Rajasthan, India; 3 Punjab Agricultural University (PAU), Ludhiana, Punjab, India; 4 Indian Council of Agricultural Research (ICAR)- Central Institute for Arid Horticulture (CIAH), Bikaner, Rajasthan, India; 5 Indian Council of Agricultural Research (ICAR)- Central Soil Salinity Research Institute (CSSRI), Karnal, Haryana, India

**Keywords:** brinjal, combining ability, diallel, environment, heterosis, stability

## Abstract

**Introduction:**

Brinjal is an essential solanaceous vegetable crop that can be cultivated globally in tropical and subtropical regions. Despite its economic significance, productivity is frequently limited by a narrow genetic base and by the under-exploitation of hybrid vigor. In order to determine superior hybrids with high yield and desirable quality traits suitable for tropical environments, the current study sought to assess the heterotic potential, combining ability, and stability of brinjal genotypes.

**Methods:**

A total of 28 F_1_ hybrids were produced by crossing eight parental genotypes in a half-diallel mating design excluding reciprocals. In Bikaner, Rajasthan, India, 36 genotypes along with their parents and the commercial check hybrid ‘Kalpataru’ were evaluated under three different sowing environments during 2019–2021 using a randomized complete block design with three replications. The growth, flowering, yield, and quality characteristics were all determined. Combining ability analysis was performed using Griffing’s method II (fixed model). Heterosis was estimated over the better parent and standard check, and the stability parameters were analyzed following the Eberhart and Russell model.

**Results:**

All traits showed significant genotype variation, indicating a high level of genetic diversity. BL-2011-219-8–1 and SL-8-PB-1-3-1–4 showed superior general combining ability for a variety of yield and quality characteristics among the parents. With better specific combining ability and heterotic performance in all environments, the hybrid Pusa Purple Long × BL-219 had the highest fruit yield (548.02 q ha^−1^). Pant Rituraj × S-324-465-2–2 and SL-8-PB-1-3-1-4 × Pant Rituraj were the two other hybrids that exhibited stable yield performance and high heterosis.

**Discussion:**

The findings indicate the involvement of both additive and non-additive gene actions in controlling brinjal yield and associated traits. The presence of additive effects suggests that selection-based breeding approaches may be effective for the improvement of certain traits in early or advanced generations, whereas the predominance of non-additive effects for some characteristics highlights the importance of heterosis breeding and hybrid development. In particular, the hybrid Pusa Purple Long × BL-219 exhibited high productivity and stability across three seasons, indicating its potential for commercial hybrid cultivation and its usefulness as a suitable parental combination in future breeding programs.

## 1 Introduction

Brinjal (*Solanum melongena* L.), an economically important vegetable crop of the Solanaceae family, is widely cultivated in tropical and subtropical regions of the world. It is the third most widely grown solanaceous vegetable in the world after tomato and potato with reference to production, domestic consumption, and export value. As reported in FAOSTAT (2024), the international production of brinjal is approximately 92.41 MMT (million metric tons), which is an increase compared with the production for the year 2021, at nearly 91.96 MMT, representing increased growth of approximately 0.49%. China retains top place, contributing over 38% (approximately 34.90 MMT) to the current global supply, with India, Egypt, Guinea, and Turkey further below it. The crop is grown on nearly 684.55 thousand hectares with a production of 13.07 MMT in India, while in Rajasthan, the area under brinjal is approximately 3.14 thousand hectares with a yield of approximately 15.09 thousand metric tons (3rd advance estimates, Government of India, 2025). Besides its economic significance, brinjal is also valued for its nutritional and medicinal properties. Saha et al. (2023) reported the fruits having low-energy density and serving as a low-calorie vegetable, which can supply valuable nutrients including proteins, carbohydrates, iron, calcium, vitamin C, niacin, and α-tocopherol. In addition, eggplant is traditionally used in folk medicine for the treatment of diseases such as asthma and bronchitis, cholera, dysuria, and some skin diseases. It has also been reported to help lower the blood cholesterol levels (Rotino et al., 2014; Meyer et al., 2014). Similar to other solanaceous crops including tomato, potato, and pepper, eggplant contains steroidal glycoalkaloids such as solasodine and solamargine, which possess several beneficial biological activities, including anticancer properties (Cham, 2012). However, excessive accumulation of these compounds may produce toxic effects and impart bitterness to the fruits, thereby reducing their palatability (Chami et al., 2003; Frary et al., 2007).

Although India is known to be a center of diversity for brinjal, much of this genetic variation has not been adequately used in breeding programs. Majority of the commercially grown varieties have a relatively wide genetic background of only 8%–12% polymorphism (Shafura et al., 2022). Moreover, plant habits such as indeterminate growth, prolonged flowering, and uneven fruit setting reduce the yield potential of brinjal (Sharma et al., 2022). Consequently, identifying suitable parental lines for hybrid development remains a major challenge for vegetable breeders because selection based on mean performance is not always advantageous for breeding improvements (Allard, 1960). Therefore, the development of high-yielding hybrids is dependent on the successful utilization of heterosis for yield and its related traits using genetically diverse and superior parental lines.

Heterosis occurs when genetically diverse parental lines are crossed to produce hybrid offspring that perform better than either parent. These hybrids are characterized by higher yield potential, early maturity, uniformity, and better-quality characteristics (Singh, 1998). Low inbreeding depression and high number of seeds per fruit in brinjal also enable the effective utilization of hybrid vigor. However, the expression of heterosis is confined only in specific hybrid combinations; hence, combining ability analysis becomes a prerequisite to derive dependable information for the selection of suitable parental lines for hybrids. This information provides breeders insights into the nature and magnitude of the genetic effects governing traits of interest. Parental lines with high general combining ability (GCA) are typically deemed useful for breeding programs, while hybrids displaying high specific combining ability (SCA) can be useful for improving desirable characteristics through hybridization. In the present study, a half-diallel mating design without reciprocals was used according to the scale methods of Griffing (1956) to examine the combining ability of parent genotypes and identify better cross combinations. Heterosis and combining ability were jointly assessed to understand the genetic control of yield and its component traits.

Despite numerous reports on heterosis and combining ability in brinjal, the majority of earlier studies were confined to single-season evaluation with limited emphasis on the stability of hybrid performance under varying seasonal conditions (Sivakumar et al., 2017; Koundinya et al., 2019). Seasonal fluctuations in temperature, relative humidity, and photoperiod can substantially influence the flowering behavior, fruit set, yield, and quality traits, thereby altering the expression of genotype × environment (G × E) interactions. Consequently, the identification of hybrids with consistent performance across growing seasons remains an important objective in brinjal breeding programs. In this context, stability models such as Eberhart and Russell and additive main effects and multiplicative interaction (AMMI) provide useful approaches for understanding the adaptability and consistency of genotypes under seasonal environmental variations (Kumawat et al., 2023; Bahadur et al., 2025). Considering the increasing demand for high-yielding and superior quality brinjal cultivars suitable for open-field cultivation, the present study was undertaken to integrate heterosis, combining ability, and stability analyses for the identification of promising hybrids across different growing seasons. A total of 28 F_1_ hybrids developed through a half-diallel mating design were evaluated to identify superior parental lines and stable hybrid combinations for yield and quality traits with potential utility in future breeding programs and commercial cultivation.

## 2 Materials and methods

### 2.1 Experimental site and climatic conditions

The present investigation was conducted during the cropping seasons of 2019–2020 and 2020–2021 at the Instructional Research Farm, College of Agriculture, Bikaner, Rajasthan, India. The experimental site is located at 28°01' N latitude and 73°22' E longitude at an elevation of 234.7 m above mean sea level and falls under the hyper-arid partially irrigated Northwestern Plain Zone of Rajasthan. The region is characterized by arid climatic conditions with extreme temperature fluctuations, low and erratic rainfall, and hot summers coupled with cold winters.

To assess the influence of seasonal climatic variations on genotype performance, the parental lines and hybrids were evaluated under three different sowing periods, which were considered as seasonal environments. These seasonal environments differed mainly with respect to prevailing temperature, relative humidity, and photoperiod during crop growth and reproductive phases, thereby generating environmental variations for studying the G × E interaction and stability performance of the hybrids.

### 2.2 Plant material

The experimental material comprised 28 F_1_ hybrids developed from eight brinjal genotypes through a half-diallel mating scheme, where reciprocal crosses were not included. In addition to these hybrids, one commercial hybrid, i.e., ‘Kalpataru’, was included as a standard check for comparison. Details of the parental genotypes, the standard check hybrid, and their sources are presented in **Table 1**. Among the parental genotypes, BL-2011-219-8-1, SL-8-PB-1-3-1-4, S-324-465-2-2, and BLW-2001-1-1–2 are advanced inbred breeding lines, whereas Pusa Purple Long, Pusa Purple Round, and Pant Rituraj are stable open-pollinated cultivars. ‘Kalpataru’ was used as the standard check for comparison.

**Table 1:** List of parents along with their sources.

Genotype	Salient features	Source
BL-2011-219-8-1	Breeding line	PAU, Ludhiana
SL-8-PB-1-3-1-4	Breeding line	PAU, Ludhiana
Pusa Purple Long	This is an early-maturing, long-fruited eggplant variety producing glossy, light purple fruits that are smooth, tender, and approximately 25–30 cm long. The crop is ready for harvesting in 100–110 days. It is suitable for both the spring and autumn planting seasons, with an average yield of approximately 27.5 t/ha, and shows moderate tolerance to shoot borer and little leaf disease.	IARI, New Delhi
BL-219	Breeding line	PAU, Ludhiana
Pusa Purple Round	This variety has medium to tall plants and produces smooth, light purple-colored fruits. It is a heavy-yielding type, with fruits having an average weight of approximately 150–200 g, making it suitable for productive cultivation.	IARI, New Delhi
Pant Rituraj	Fruits are round with attractive purple color with fewer seeds. Gives average yield of 160 q/acre.	GBPUAT Pantnagar
S-324-465-2-2	Breeding line	PAU, Ludhiana
BLW-2001-1-1-2	Breeding line	PAU, Ludhiana
BL-2011-219-8-1	Breeding line	PAU, Ludhiana
‘Kalpataru’ (control)	This hybrid eggplant produces attractive purple fruits with white stripes and an oval shape, weighing 70–80 g. Plants are vigorous with high yield potential and a spiny green calyx. Strong rejuvenation capacity allows repeated harvesting, making it suitable for curry preparation and processing.	Mahyco Pvt. Ltd.

### 2.3 Development of F_1_ hybrids

During the first year (Mar-Jun 2019), the eight parental lines were intercrossed according to a half-diallel mating system with reciprocals excluded. Initially, seeds of the parent lines were raised in pro trays filled with a potting mixture of cocopeat, perlite, and vermiculite at a ratio of 3:1:1. The seedlings were maintained in the nursery and subsequently transplanted to the main field after 3–4 weeks of growth. The seedlings were transplanted under three different environmental conditions. In the first environment, the crop was sown during the first week of July and harvested in the fourth week of October. In the second environment, sowing was carried out in the first week of September and the final harvest completed in the third week of November. In the third environment, the crop was sown in the first week of November and harvested at the end of January to the first week of February. These three sowing periods exposed the crop to different temperature and humidity regimes during the vegetative and reproductive growth stages, thereby generating seasonal environmental variations for the evaluation of genotype performance and stability. The parental lines were selfed in the following season to obtain pure seeds. Fully matured fruits from both the parental lines and the hybrids were collected, and the seeds were extracted and dried under sunlight before storage for subsequent use.

### 2.4 Mainland preparation and transplantation

Proper plowing and leveling of the land was done to make a fine tilth for good establishment of brinjal. For the experimental layout, land was manually furrowed with a marker maintaining 60-cm row spacing and individual plots of 3.0 m × 3.6 m area, and transplantation followed three environmental windows across the seasons from 2020 to 2021 using accurate plant-to-plant spacing of 40 cm. Each genotype was represented by 30 plants, and data were taken from 10 plants in the experimental unit of 7.2 m^2^. Each sowing period was treated as an independent seasonal environment and evaluated separately using a randomized complete block design (RCBD) with three replications. Subsequently, pooled analysis across seasonal environments was performed for stability analysis.

### 2.5 Crop management practices

Recommended agronomic practices for brinjal cultivation were uniformly followed throughout the experimental period (Dhaliwal, 2017). Fertilizers were applied as per regional recommendations, and irrigation was scheduled according to crop requirements and prevailing climatic conditions. Weed management involved manual weeding and intercultural operations, while major insect pests were managed using recommended plant protection measures.

### 2.6 Recording of observations

Data were collected on various phenotypic traits: plant height at 90 days after transplanting (DAT, in centimeters), number of primary branches at 90 DAT (number per plant), days to first flowering (days), days to 50% flowering (days), number of flowers per cluster (number), number of fruits per cluster (number), fruit setting (percent), days to first fruit harvest (days), number of fruits per plant (number), fruit length (in centimeters), fruit diameter (in centimeters), fruit yield per plant (in grams), average fruit weight (in grams), and fruit yield per hectare (in quintals per hectare). These parameters were taken from five randomly selected plants in each replication. Moreover, the iron content (in milligrams per 100 g) in fruit samples was estimated from diacid-digested samples using ICP-MS (model NexION 300X; Perkin Elmer, Springfield, IL, USA), and the concentration was calculated using the dilution factor and expressed in milligrams per 100 g. Total soluble solids (°Brix) were measured from fruit pulp using a hand refractometer. Titratable acidity (in percent) was determined by titrating fruit juice against 0.1 N NaOH using a phenolphthalein indicator. The anthocyanin content (in percent) was estimated from dried peel powder extracted with ethanol, and absorbance was recorded at 525 nm to determine the anthocyanin concentration.

### 2.7 Estimation of heterosis

Heterosis was calculated as the percentage difference in the mean performance of the F_1_ hybrids relative to the better parent and the standard check hybrid ‘Kalpataru’. The assessment of heterotic response was performed according to the methods proposed by Shull (1908) and later described by Fonseca and Patterson (1968). The following expressions were used to estimate heterosis:

Mid parent heterosis (MPH) = $(F_{1}-\text{MP})/\text{MP }$ × 100.

Better parent heterosis (BPH) = $(F_{1}-BP)/BP$ × 100.

Standard heterosis (SH) = $(F_{1}-SC)/SC$ × 100.

where F_1_ denotes the mean performance of the hybrid, BP represents the mean value of the superior parent, and SC indicates the mean performance of the standard check hybrid.

The significance of the heterosis values was determined using the least significant difference (LSD) test. LSD was calculated using the following formula:


LSD=SE(d)×t

where SE(*d*) = ± $\sqrt{2\text{Me}/r}$. Me represents the error mean square obtained from the analysis of variance (ANOVA) of the RCBD involving parents, F_1_ hybrids, and the standard check, while *r* refers to the number of replications. The significance of the differences was tested at the *p* ≤ 0.05 and *p* ≤ 0.01 probability levels.

### 2.8 Combining ability analysis

The combining ability among the parental genotypes was estimated according to the diallel analysis procedure proposed by Griffing (1956). In the present study, method II with model I (fixed effects) was adopted, which considers the parents and a single set of F_1_ hybrids while excluding reciprocal crosses. ANOVA was carried out using the following statistical model:


$$X_{ij}=\mu +g_{i}+g_{j}+S_{ij}+\frac{1}{bc}\sum_{k}\sum_{l}e_{ijkl}$$

where *i* and *j* range from 1 to *p* (number of parental lines), *k* ranges from 1 to *b* (number of replications or blocks), and *l* ranges from 1 to *c* (number of observations recorded per plot). In this model, *X_ij_* represents the average performance of the hybrid derived from the *i*th and *j*th parents across replications and observations. The term *μ* denotes the overall mean of the experiment. The parameters *g_i_* and *g_j_* indicate the GCA effects associated with the *i*th and *j*th parents, respectively, while *S_ij_* represents the SCA effect of the cross between the two parents, assuming that *S_ij_* = *S_ji_*. The term *e_ijkl_* accounts for the experimental error attributed to environmental variations during the observation.

### 2.9 Stability analysis

To evaluate the performance of the genotypes across different seasonal environments, stability parameters were computed using the model proposed by Eberhart and Russell (1966).


$$Y_{ij}=\mu_{i}+\beta_{i}I_{j}+\delta_{ij}$$

where *Y_ij_* represents the mean performance of the *i*th genotype in the *j*th environment, *μ_i_* denotes the mean of the *i*th genotype over all environments, $\beta_{i}\;$is the regression coefficient of the *i*th genotype indicating its linear response to environmental changes, *δ_ij_* refers to the deviation from regression, and $I_{j}\;$is the environmental index calculated as the mean of all genotypes in the *j*th environment minus the grand mean. The stability of a genotype was assessed based on three parameters: the mean ( $\bar{x}$), the regression coefficient ( $b_{i}$), and the mean square deviation from regression (*S*^2^*d_i_* = 0). A genotype is considered stable when it exhibits high mean performance, a regression coefficient close to unity (*b_i_* = 1), and a non-significant deviation from regression (*S*^2^*d_i_* = 0). The significance of the variance due to genotypes, environments, and G × E interactions was examined against the pooled error, whereas environment (linear) and G × E (linear) effects were assessed against the pooled deviation.

### 2.10 Statistical analysis

The collected data from the parental lines and the F_1_ hybrids were subjected to ANOVA to determine the presence of significant genetic variation among the genotypes. Statistical analysis of the experiment was carried out following a RCBD with three replications in order to evaluate the variations among parents and the F_1_ hybrids and the contrast between the parental lines and hybrids. The significance of the variation among crosses was assessed using two-way ANOVA following the statistical procedures described by Ihaka and Gentleman (1996).

## 3 Results

### 3.1 Statistical analysis of variance for the experiment

ANOVA was performed for 14 phenotypic traits and four quality parameters: plant height at 90 DAT (in centimeters), number of primary branches at 90 DAT (per plant), days to first flowering, days to 50% flowering, number of flowers per cluster (per cluster), number of fruits per cluster (per cluster), fruit set (in percent), days to first fruit harvest, number of fruits per plant (per plant), fruit length (in centimeters), fruit diameter (in centimeters), average fruit weight (in grams), fruit yield per plant (in grams), fruit yield per hectare (in quintals per hectare), iron content (in milligrams per 100 g), total soluble solids (TSS, °Brix), titratable acidity (in percent), and anthocyanin content (in percent). ANOVA showed significant differences among genotypes for all traits evaluated, indicating significant genetic variability (**Table 2**). **Table 3** represents the per se performance of hybrids and parents, while **Supplementary Tables 1**–**5** indicates the results of GCA, SCA, heterosis, heterobeltiosis, and G × E interaction analysis of the 28 hybrids.

**Table 2:** Analysis of variance for the experimental design showing the mean squares for different traits in brinjal and the estimation of genetic components of variation.

Source	*df*	Plant height (cm) at 90 DAT	No. of branches at 90 DAT	Days to first flowering	Days to 50% flowering	No. of flowers per cluster	No. of fruits per cluster	Fruit setting (%)	Days to first fruit harvest	No. of fruits per plant	Fruit length (cm)	Fruit diameter (cm^2^)	Fruit yield per plant (g)	Average fruit weight (g)	Fruit yield (q ha^−1^)	Iron (mg 100g^−1^)	Total soluble solid (°Brix)	Acidity (%)	Anthocyanin (%)
Environment	2	1,398.73**	12.95**	198.72**	232.61**	38.71**	26.86**	1,182.78**	229.78**	191.41**	38.80**	38.24**	4,113,461.0**	11,397.83**	714,142.5**	0.012**	3.418**	0.027**	212.251**
Blocks within environment	6	33.11	0.46	6.73	7.65	0.09	0.14	34.57	8.63	0.72	0.42	0.31	9,587.03	56.42	1,664.415	0	0.142	0	3.256
Genotypes	35	395.32**	6.95**	116.82**	129.89**	1.37**	1.97**	417.28**	150.55**	15.45**	12.87**	7.09**	222,695.40**	676.28**	38,662.39**	0.004**	1.585**	0.003**	38.700**
Parents	7	156.722**	2.35**	123.42**	137.53**	0.46**	0.66**	185.43**	159.61**	5.00**	6.68**	5.50**	222,619.50**	1,015.22**	38,649.22**	0.001*	0.402*	0.003**	9.665*
Hybrids	27	392.36**	7.058**	117.96**	131.111**	1.39**	2.01**	383.36**	151.93**	15.72**	13.120**	7.75**	197,826.50**	586.82**	34,344.87**	0.005**	1.631**	0.004**	40.347**
Parents *vs*. hybrids	1	2,145.54**	36.18**	40.01**	43.23**	7.07**	10.06**	2,956.16**	49.70**	81.43**	49.42**	0.62	894,687.80**	719.25**	155,327.80**	0.024**	8.617**	0.001*	197.473**
Genotypes × environment	70	3.265	0.06	4.99	5.39	0.012	0.035	6.871	6.087	0.14	0.132	0.23	5,284.93	48.341	917.52	0	0.052	0	1.468
Parent × environment	14	2.704	0.04	4.03	4.57	0.01	0.016	3.242	5.38	0.11	0.12	0.18	5,396.15	35.244	936.83	0	0.024	0	0.734
Hybrids × environment	54	3.509	0.06	4.81	5.09	0.013	0.037	7.993	5.624	0.15	0.14	0.20	4,189.34	48.422	727.32	0	0.061	0	1.709
Parent *vs*. hybrids × environment	2	0.596	0.11	16.97*	19.29*	0.023	0.094	1.995	23.54*	0.04	0.04	0.76 *	34,087.72	137.819*	5,918.01*	0	0	0	0.074
Error	210	25.631	0.4	5.541	6.15	0.08	0.119	30.557	7.12	0.92	0.95	0.25	8,339.95	45.29	1,447.91	0	0.178	0	4.302

*DAT*, days after transplanting.

*p < 0.05, **p < 0.01.

**Table 3:** Comparative per se performance of the growth, yield, and biochemical characteristics of brinjal.

SN	Cross	PH	NB	DFF	D50%F	NFLC	NFC	FS	DFH	NFPP	FL	FD	FWT	FE	TSS	Acidity	ANTH
1.	BL-2011-219-8-1 × SL-8-PB-1-3-1-4	73.99	9.80	59.02	63.04	6.23	4.93	79.17	69.73	14.69	14.49	4.77	79.14	0.28	6.55	0.26	20.59
2.	BL-2011-219-8-1 × Pusa Purple Long	71.85	9.20	58.31	62.30	5.96	4.62	76.74	68.92	13.60	13.50	4.63	72.68	0.28	6.58	0.26	20.78
3.	BL-2011-219-8-1 × BL-219	58.43	7.55	65.91	70.29	5.23	3.74	62.26	77.53	11.12	10.89	6.23	78.14	0.25	5.94	0.3	17.58
4.	BL-2011-219-8-1 × Pusa Purple Round	63.23	8.14	67.56	72.03	5.48	4.04	67.37	79.42	11.97	11.24	6.57	83.99	0.27	6.31	0.31	19.39
5.	BL-2011-219-8-1 × Pant Rituraj	60.15	7.76	67.36	71.82	5.33	3.86	64.02	79.18	11.43	11.20	6.53	83.49	0.25	6.04	0.31	18.05
6.	BL-2011-219-8-1 × S-324-465-2-2	59.06	7.63	66.39	70.80	5.27	3.79	62.83	78.08	11.22	10.99	6.33	79.84	0.25	5.98	0.3	17.76
7.	BL-2011-219-8-1 × BLW-2001-1-1-2	57.18	7.40	64.85	69.18	5.16	3.66	60.79	76.33	10.90	10.67	6.00	74.49	0.24	5.85	0.29	17.1
8.	SL-8-PB-1-3-1-4 × Pusa Purple Long	70.22	9.33	59.14	63.17	6.02	4.69	74.97	69.85	13.71	13.50	4.80	75.29	0.29	6.79	0.26	21.73
9.	SL-8-PB-1-3-1-4 × BL-219	59.57	7.69	63.70	67.97	5.29	3.81	63.39	75.14	11.32	11.10	5.76	74.01	0.25	6.03	0.29	18.01
10.	SL-8-PB-1-3-1-4 × Pusa Purple Round	69.43	8.90	57.76	61.72	5.83	4.46	74.10	68.30	13.08	12.09	4.51	64.76	0.29	6.67	0.24	21.21
11.	SL-8-PB-1-3-1-4 × Pant Rituraj	64.65	8.31	64.60	68.92	5.57	4.15	68.91	76.05	12.23	12.01	5.95	81.84	0.27	6.41	0.29	19.78
12.	SL-8-PB-1-3-1-4 × S-324-465-2-2	57.80	7.47	65.37	69.73	5.19	3.70	61.47	76.93	11.01	10.78	6.11	76.11	0.24	5.89	0.3	17.34
13.	SL-8-PB-1-3-1-4 × BLW-2001-1-1-2	62.86	8.09	64.92	69.25	5.47	4.03	66.85	76.41	11.91	10.68	6.02	74.52	0.26	6.25	0.29	19.13
14.	Pusa Purple Long × BL-219	67.24	9.18	68.67	73.20	5.96	4.60	71.72	80.66	13.58	12.81	6.78	97.39	0.3	6.95	0.31	22.61
15.	Pusa Purple Long × Pusa Purple Round	58.28	7.55	65.49	69.72	5.23	3.72	61.96	76.85	10.93	10.70	6.11	76.76	0.25	5.94	0.3	17.66
16.	Pusa Purple Long × Pant Rituraj	56.34	7.31	64.48	68.78	5.11	3.57	59.87	75.86	10.74	10.62	5.92	73.78	0.24	5.8	0.29	16.82
17.	Pusa Purple Long × S-324-465-2-2	52.28	6.79	65.79	70.17	4.90	3.34	55.46	77.39	10.02	10.79	6.20	77.20	0.22	5.48	0.3	15.36
18.	Pusa Purple Long × BLW-2001-1-1-2	50.20	6.55	64.03	68.31	4.79	3.20	53.20	75.47	9.65	10.42	5.83	71.41	0.21	5.33	0.29	14.6
19.	BL-219 × Pusa Purple Round	53.98	7.00	62.49	66.70	4.99	3.45	57.30	73.76	10.32	10.09	5.51	65.81	0.23	5.6	0.28	15.89
20.	BL-219 × Pant Rituraj	56.76	7.35	65.37	69.73	5.13	3.62	60.55	76.90	10.93	10.70	6.11	75.69	0.24	5.82	0.3	16.74
21.	BL-219 × S-324-465-2-2	56.88	7.36	64.68	69.00	5.15	3.64	60.46	76.24	10.79	10.56	5.97	73.29	0.24	5.82	0.29	16.88
22.	BL-219 × BLW-2001-1-1-2	45.73	6.00	72.35	77.08	4.54	2.91	63.01	84.82	8.85	8.61	8.58	84.31	0.22	5.36	0.33	14.66
23.	Pusa Purple Round × Pant Rituraj	53.21	6.91	71.37	76.17	4.95	3.40	56.47	83.85	10.19	9.95	8.40	93.82	0.23	5.55	0.33	15.49
24.	Pusa Purple Round × S-324-465-2-2	58.22	7.52	65.97	70.36	5.21	3.72	61.92	77.60	11.06	10.83	6.24	77.73	0.25	5.93	0.3	17.5
25.	Pusa Purple Round × BLW-2001-1-1-2	61.61	7.94	68.78	73.32	5.40	3.95	65.60	80.79	11.65	11.43	6.83	88.41	0.26	6.18	0.31	18.67
26.	Pant Rituraj × S-324-465-2-2	63.02	8.11	70.30	74.92	5.48	4.04	67.14	82.62	11.97	11.75	7.15	94.52	0.27	6.29	0.32	19.42
27.	Pant Rituraj × BLW-2001-1-1-2	53.17	6.90	62.88	67.11	4.94	3.40	56.43	74.08	10.41	10.17	5.59	67.28	0.23	5.54	0.28	15.59
28.	S-324-465-2-2 × BLW-2001-1-1-2	59.95	7.73	67.49	71.84	5.31	3.84	63.80	79.19	11.36	11.13	6.53	83.13	0.25	6.06	0.31	18.15
29.	BL-2011-219-8-1	56.27	7.29	66.17	70.56	5.12	3.59	59.80	77.84	10.62	11.40	6.28	82.54	0.24	5.78	0.3	16.77
30.	SL-8-PB-1-3-1-4	50.05	6.53	61.44	65.58	4.78	3.19	53.02	72.45	9.62	10.39	5.28	66.50	0.21	5.32	0.28	14.66
31.	Pusa Purple Long	53.53	6.95	64.49	68.68	4.96	3.42	56.82	75.82	10.24	11.02	5.90	75.14	0.23	5.57	0.29	15.72
32.	BL-219	55.52	7.19	68.94	73.49	5.07	3.55	58.98	80.98	10.60	10.37	6.86	82.24	0.23	5.72	0.32	16.81
33.	Pusa Purple Round	56.87	7.36	70.09	74.69	5.15	3.64	60.45	82.28	10.84	10.61	7.11	85.74	0.24	5.82	0.32	16.99
34.	Pant Rituraj	45.62	5.98	60.42	64.51	4.53	2.91	48.23	71.31	8.80	8.56	5.07	53.58	0.22	5.45	0.27	15.13
35.	S-324-465-2-2	52.82	6.86	66.66	71.09	4.93	3.38	56.05	78.39	10.12	9.88	6.38	73.26	0.22	5.52	0.3	15.45
36.	BLW-2001-1-1-2	58.45	7.55	70.00	74.60	5.23	3.74	62.17	82.18	11.12	9.90	7.09	80.56	0.25	5.94	0.32	17.59
37.	‘Kalpataru’	61.55	7.93	69.66	74.24	5.40	3.95	65.54	81.79	11.55	10.33	7.02	82.61	0.26	6.18	0.32	18.75
38.	S. Em	1.67	0.21	0.79	0.83	0.09	0.11	1.82	0.90	0.32	0.32	0.17	2.24	0.01	0.17	0.01	0.84
39.	CD@5%	4.65	0.58	2.20	2.32	0.26	0.32	5.08	2.49	0.88	0.90	0.46	6.23	0.03	0.48	0.02	2.32

PH, plant height; NB, number of branches per plant; DFF, days to first flowering; D50%F, days to 50% flowering; NFLC, number of flowers per flower cluster; NFC, number of fruits per cluster; FS, fruit set (in percent); DFH, days to first harvest; NFPP, number of fruits per plant; FL, fruit length; FD, fruit diameter; FWT, fruit weight; FYPP, fruit yield per plant; FE, iron content; TSS, total soluble solids; ACD, acidity; ANTH, anthocyanin content.

### 3.2 Evaluation of parental mean performance and general combining ability

**Figure 1:**
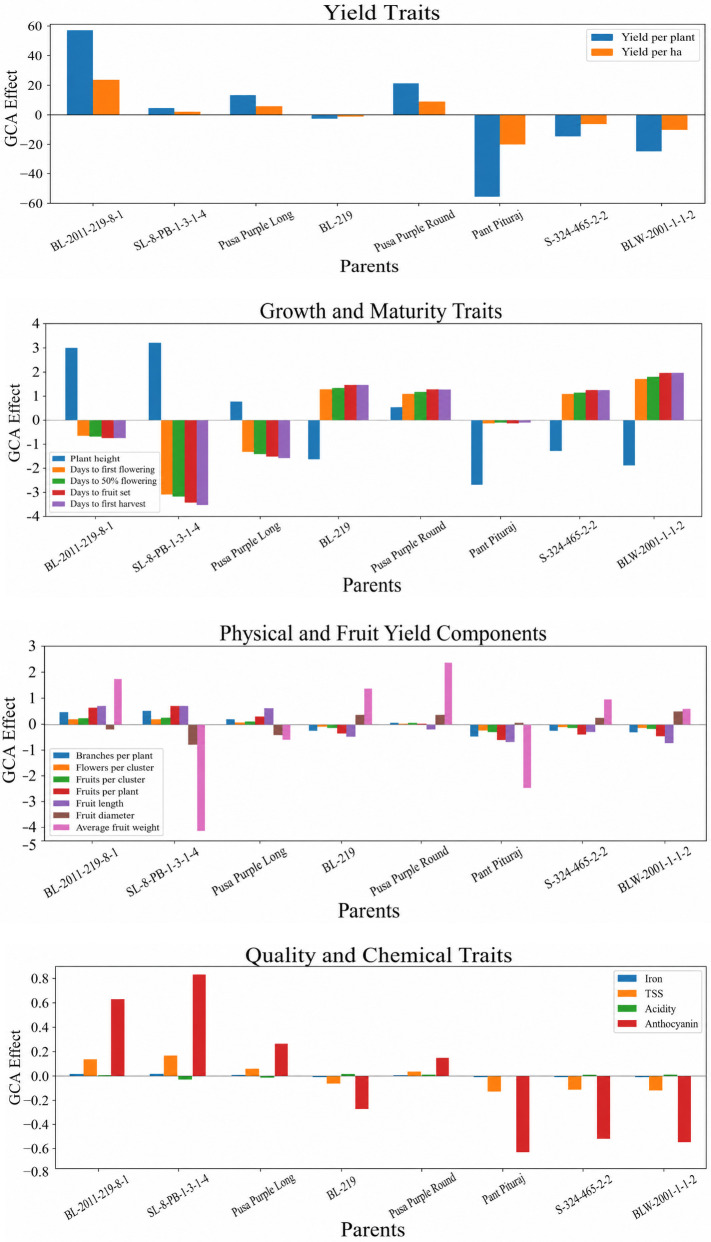
Graphical representation of the percentage general combining ability (GCA) of eight parents (BL-2011-219-8-1, SL-8-PB-1-3-1-4, Pusa Purple Long, BL-219, Pusa Purple Round, Pant Rituraj, S-324-465-2-2, and BLW-2001-1-1-2) for the yield traits, growth and maturity traits, physical and fruit yield components, and quality and chemical traits.

The pooled observations and GCA estimates for all eight brinjal genotypes are shown in **Figure 1** and **Supplementary Table 1**. Highly significant differences in the GCA effects were found among the parents for all growth, flowering, yield, and quality traits, indicating the involvement of additive gene action. Plant height at 90 DAT showed GCA effects from −2.92 to 3.53 in different environments, with BL-2011-219-8–1 and SL-8-PB-1-3-1–4 consistently showing significant positive effects under pooled analysis, with GCA values ranging from −2.75 (Pant Rituraj) to 3.25 (SL-8-PB-1-3-1-4), confirming the latter as the best general combiner for plant height. A similar trend was observed for the number of branches at 90 DAT, where the GCA effects ranged from −0.37 to 0.50 and the pooled estimates varied from −0.26 (BLW-2001-1-1-2) to 0.44 (SL-8-PB-1-3-1-4), indicating the superiority of BL-2011-219-8–1 and SL-8-PB-1-3-1–4 for improving branching ability. For earliness traits, negative GCA effects are considered desirable. Days to first flowering ranged from −3.50 to 1.89, while days to 50% flowering varied from −3.67 to 1.93. In both traits, SL-8-PB-1-3-1–4 recorded the highest negative pooled GCA effects (−3.10 and −3.25, respectively), followed by BL-2011-219-8–1 and Pusa Purple Long, indicating their suitability for the development of early flowering genotypes. Similar results were observed for days to first fruit harvest, where the GCA effects ranged from −3.84 to 2.08. SL-8-PB-1-3-1–4 recorded the highest negative pooled estimate (−3.49), suggesting its usefulness for the breeding of early maturing hybrids. Reproductive traits also showed considerable variability among the parents. The number of flowers per cluster ranged from −0.18 to 0.23, the number of fruits per cluster from −0.24 to 0.29, and the fruit set percentage from −3.49% to 3.33%. BL-2011-219-8–1 and SL-8-PB-1-3-1–4 consistently exhibited positive GCA effects across environments for these traits. Similarly, the pooled GCA effects for the number of fruits per plant ranged from −0.53 to 0.70, with SL-8-PB-1-3-1–4 recording the highest positive estimate (0.65), followed by BL-2011-219-8–1 and Pusa Purple Long, indicating their effectiveness in improving fruit number and reproductive efficiency.

In addition to the vegetative traits, significant variation was also observed for fruit morphology, yield, and quality traits. The GCA effects for fruit length ranged from −0.63 to 0.71. Pooled analysis indicated that BL-2011-219-8-1 (0.68) was the best general combiner, followed by SL-8-PB-1-3-1–4 and Pusa Purple Long. Fruit diameter ranged from −0.78 to 0.45, with BL-219 and Pusa Purple Round showing favorable pooled GCA effects. The average fruit weight ranged from −5.13 to 3.34 g. Pusa Purple Round had the highest pooled positive GCA effect (2.32), followed by BL-2011-219-8-1, indicating their usefulness for the development of hybrids with heavier fruits. The GCA effects for fruit yield per plant ranged widely from −54.18 to 70.30 across environments and for fruit yield per hectare from −22.58 to 29.29. BL-2011-219-8–1 exhibited positive GCA effects in all environments and also recorded the highest pooled estimates for fruit yield per plant (57.12) and fruit yield per hectare (23.80), thus emerging as the best general combiner for yield traits. The quality parameters also showed significant variations. Iron content ranged from −0.01 to 0.01, with positive pooled GCA effects for SL-8-PB-1-3-1–4 and BL-2011-219-8-1, indicating their utility for micronutrient enhancement. TSS ranged from −0.15 to 0.18, with the highest positive pooled GCA effect (0.171) recorded in SL-8-PB-1-3-1-4, followed by BL-2011-219-8-1. Negative GCA effects are desirable for titratable acidity, and SL-8-PB-1-3-1–4 recorded the highest negative pooled estimate (−0.017), followed by Pusa Purple Long. The anthocyanin content varied from −0.81 to 1.00, with SL-8-PB-1-3-1–4 showing significant positive GCA effects in all environments and the highest pooled value (0.870). BL-2011-219-8–1 also showed favorable pooled effects, suggesting its potential to improve nutraceutical quality in brinjal.

### 3.3 Specific combining ability effects of cross combinations

**Figure 2:**
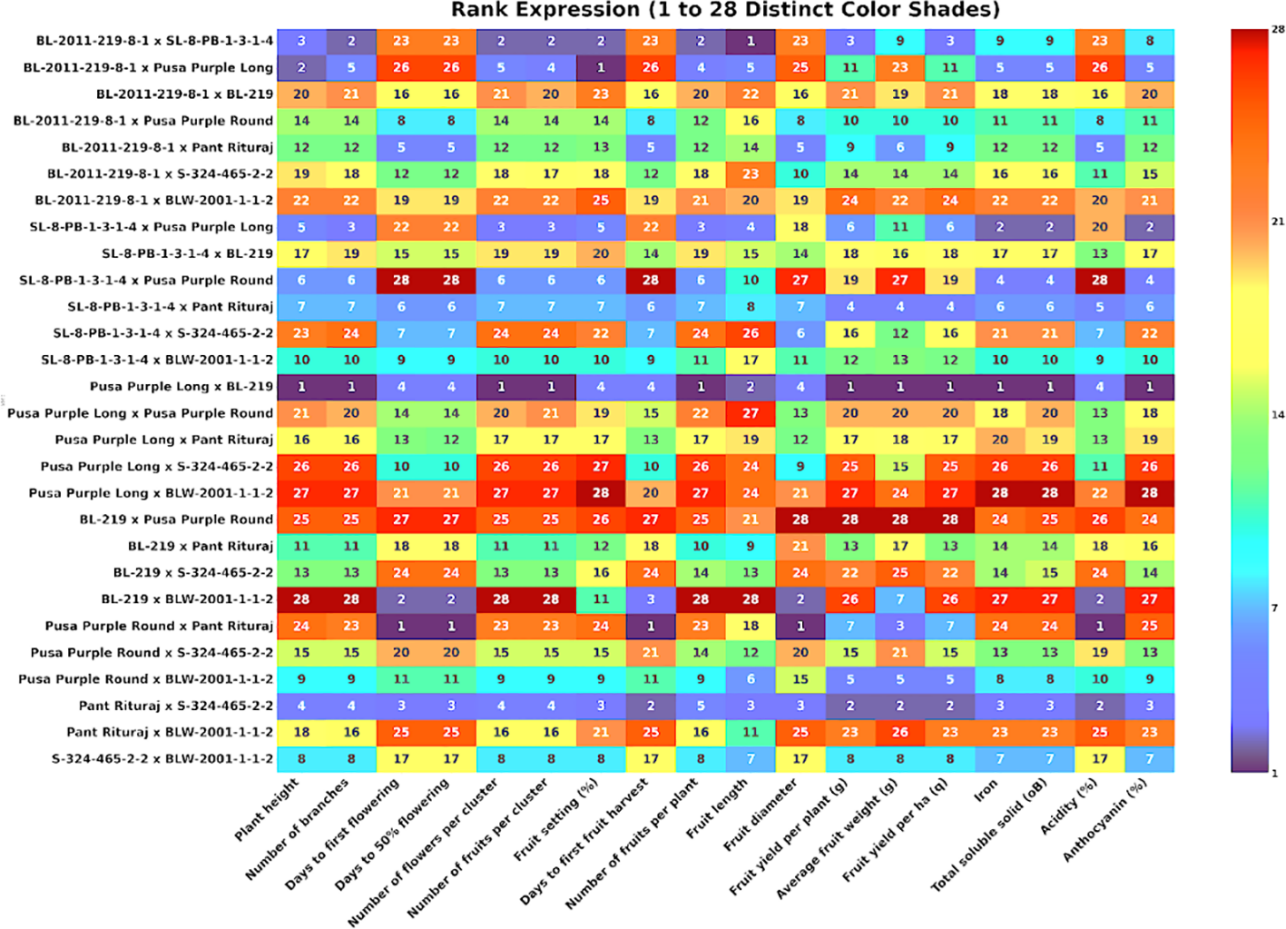
Heatmap representation of the specific combining ability (SCA) effects for 18 growth, yield, and nutritional traits among various hybrids.

The SCA effects revealed significant variations among the cross combinations for all the growth, yield, and quality traits studied (**Figure 2**; **Supplementary Table 2**), indicating the predominance of non-additive gene action. For vegetative traits, plant height at 90 DAT ranged from −9.22 to 9.69, with Pusa Purple Long × BL-219 recording the highest positive SCA effect (9.69**), followed by BL-2011-219-8-1 × Pusa Purple Long (9.62**), BL-2011-219-8-1 × SL-8-PB-1-3-1-4 (9.25**), and Pant Rituraj × S-324-465-2-2 (8.65**). Similarly, the number of branches at 90 DAT ranged from −1.15 to 1.62, where Pusa Purple Long × BL-219 (1.62**), BL-2011-219-8-1 × SL-8-PB-1-3-1-4 (1.39**), SL-8-PB-1-3-1-4 × Pusa Purple Long (1.15**), and Pant Rituraj × S-324-465-2-2 (1.09**) exhibited significant positive SCA effects. For earliness traits, negative SCA effects are considered desirable. Days to first flowering ranged from −5.64 to 4.98, while days to 50% flowering ranged from −5.94 to 5.35. For both traits, SL-8-PB-1-3-1-4 × Pusa Purple Round and BL-219 × Pusa Purple Round recorded the most desirable negative SCA effects. Similarly, days to first fruit harvest varied from −6.41 to 5.76, where SL-8-PB-1-3-1-4 × Pusa Purple Round (−6.41**) and BL-219 × Pusa Purple Round (−5.92**) were identified as superior combinations for early maturity. Reproductive traits also exhibited substantial variations among hybrids. The number of flowers per cluster ranged from −0.51 to 0.72, and significant positive SCA effects were observed in Pusa Purple Long × BL-219 (0.72**), BL-2011-219-8-1 × SL-8-PB-1-3-1-4 (0.62**), and Pant Rituraj × S-324-465-2-2 (0.49**). The number of fruits per cluster ranged from −0.62 to 0.86, with the same hybrids recording superior positive effects. The fruit set percentage varied from −8.89% to 10.78%, where BL-2011-219-8-1 × Pusa Purple Long (10.78**), BL-2011-219-8-1 × SL-8-PB-1-3-1-4 (10.47**), and Pant Rituraj × S-324-465-2-2 (9.71**) showed the highest positive SCA effects. Similarly, the number of fruits per plant ranged from −1.71 to 2.44, with Pusa Purple Long × BL-219 (2.44**), BL-2011-219-8-1 × SL-8-PB-1-3-1-4 (2.30**), and Pant Rituraj × S-324-465-2-2 (1.60**) identified as superior combinations.

Significant variations were also observed for the fruit, yield, and quality traits. Fruit length ranged from −1.44 to 2.16, where BL-2011-219-8-1 × SL-8-PB-1-3-1–4 recorded the highest positive SCA effect (2.16**), followed by Pusa Purple Long × BL-219 (1.62**) and Pant Rituraj × S-324-465-2-2 (1.53**). Fruit diameter ranged from −1.27 to 1.91, with Pusa Purple Round × Pant Rituraj (1.91**), BL-219 × BLW-2001-1-1-2 (1.69**), and Pant Rituraj × S-324-465-2-2 (0.77**) exhibiting favorable SCA effects. The fruit yield per plant showed wide variations ranging from −215.03 to 437.64, and the hybrids Pusa Purple Long × BL-219 (437.64**), Pant Rituraj × S-324-465-2-2 (328.88**), and BL-2011-219-8-1 × SL-8-PB-1-3-1-4 (227.33**) recorded the highest positive SCA effects. A similar trend was observed for fruit yield per hectare, where the SCA effects ranged from −89.59 to 182.34, with Pusa Purple Long × BL-219 (182.34**), Pant Rituraj × S-324-465-2-2 (137.03**), and BL-2011-219-8-1 × SL-8-PB-1-3-1-4 (94.72**) identified as superior hybrids. Average fruit weight ranged from −15.59 to 18.78, and significant positive SCA effects were observed in Pusa Purple Long × BL-219 (18.78**), Pant Rituraj × S-324-465-2-2 (18.11**), and Pusa Purple Round × Pant Rituraj (15.97**). Among the quality traits, iron content ranged from −0.030 to 0.054, with the highest positive SCA effect observed in Pusa Purple Long × BL-219 (0.054**), followed by SL-8-PB-1-3-1-4 × Pusa Purple Long (0.033**) and Pant Rituraj × S-324-465-2-2 (0.030**). TSS ranged from −0.563 to 1.010. Pusa Purple Long × BL-219 (1.010**) and SL-8-PB-1-3-1-4 × Pusa Purple Long (0.625**) showed better performance. Titratable acidity ranged from −0.042 to 0.027, and desirable negative SCA effects were observed in SL-8-PB-1-3-1-4 × Pusa Purple Round (−0.042**) and BL-219 × Pusa Purple Round (−0.026**). Anthocyanin content ranged from −2.724 to 4.992, with the highest positive SCA effects observed in Pusa Purple Long × BL-219 (4.992**), SL-8-PB-1-3-1-4 × Pusa Purple Long (2.999**), and Pant Rituraj × S-324-465-2-2 (2.957**), indicating scope for the improvement of nutraceutical quality in brinjal hybrids.

### 3.4 Identification of cross combinations on the basis of per se performance

**Figure 3:**
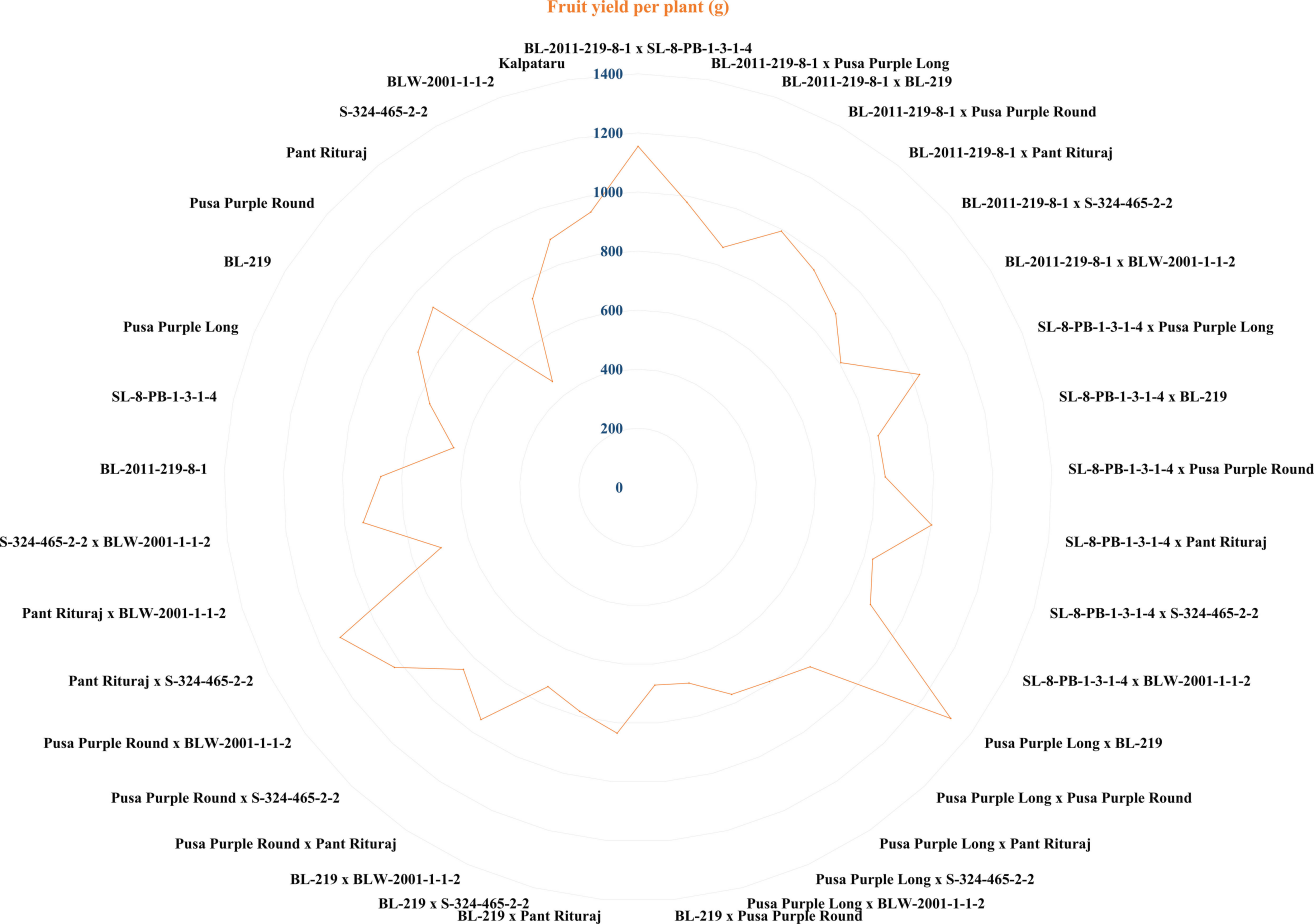
Illustration of the comparative mean performance of the parental lines and their F_1_ hybrids for fruit yield per plant (per gram).

Fruit yield is the ultimate objective of any crop improvement program. Therefore, the mean performance of the 28 F_1_ hybrids was evaluated for 13 yield-related traits and four quality parameters (**Table 3**). The mean comparisons of the parents and hybrids for fruit yield per plant are presented in **Figure 3**. Considerable variations were observed among the hybrids for yield and its contributing traits. The fruit yield per plant ranged between 684.3 and 1,315.2 g, with hybrid Pusa Purple Long × BL-219 producing the highest yield (1,315.2 g) and Pusa Purple Long × BLW-2001-1-1–2 giving the lowest yield (684.3 g). The yield superiority was mainly associated with higher number of fruits per plant and greater average fruit weight. The number of fruits per plant varied from 8.85 to 14.69, and BL-2011-219-8-1 × SL-8-PB-1-3-1–4 produced the maximum number of fruits (14.69), whereas BL-219 × BLW-2001-1-1–2 recorded the minimum value (8.85). The average fruit weight ranged from 64.76 to 97.39 g, with Pusa Purple Long × BL-219 exhibiting the highest fruit weight (97.39 g), while SL-8-PB-1-3-1-4 × Pusa Purple Round recorded the lowest value (64.76 g). Fruit diameter ranged from 4.51 to 8.58 cm, and the maximum diameter was observed in BL-219 × BLW-2001-1-1-2 (8.58 cm), followed by Pusa Purple Round × Pant Rituraj (8.40 cm). Fruit length ranged from 8.61 to 14.49 cm, with BL-2011-219-8-1 × SL-8-PB-1-3-1–4 recording the maximum fruit length (14.49 cm). Earliness traits also exhibited considerable variations among hybrids. Days to first flowering ranged from 57.76 to 72.35 days, days to 50% flowering from 61.72 to 77.08 days, and days to first fruit harvest from 68.30 to 84.82 days. In all these traits, SL-8-PB-1-3-1-4 × Pusa Purple Round was identified as the earliest hybrid, while BL-219 × BLW-2001-1-1–2 was the late hybrid.

Considerable variations were also observed for the reproductive and quality traits among the hybrids. BL-2011-219-8-1 × SL-8-PB-1-3-1–4 recorded the highest number of flowers per cluster (6.23), fruits per cluster (4.93), fruit set percentage (79.17%), plant height (73.99 cm), and number of branches per plant (9.80), indicating its superiority for the yield-contributing traits. In contrast, BL-219 × BLW-2001-1-1–2 recorded the lowest values for the majority of these characteristics. Among the quality traits, TSS ranged from 5.33 to 6.95°Brix, with Pusa Purple Long × BL-219 recording the highest TSS content. Iron content ranged from 0.21 to 0.30, and the highest iron content was also recorded in Pusa Purple Long × BL-219. Titratable acidity ranged from 0.24 to 0.33, where SL-8-PB-1-3-1-4 × Pusa Purple Round exhibited the lowest acidity, indicating better flavor balance. Anthocyanin content ranged from 14.60 to 22.61 mg 100 g^−1^, and the hybrid Pusa Purple Long × BL-219 recorded the highest anthocyanin content, demonstrating its superiority for nutraceutical quality. The standard check hybrid ‘Kalpataru’ exhibited intermediate values for all quality parameters of 0.26 (iron content), 6.18 (TSS), 0.32 (acidity), and 18.75 (anthocyanins).

### 3.5 Estimation of standard heterosis and heterobeltiosis

The estimates of heterobeltiosis (hb) and standard heterosis (sh) over the commercial check ‘Kalpataru’ for the yield and quality traits are presented in **Supplementary Table 3**. Considerable heterosis was observed among the hybrids for several economically important traits. For the vegetative traits, BL-2011-219-8-1 × SL-8-PB-1-3-1–4 recorded the highest significant positive hb and hs for plant height (31.48% and 20.21%, respectively) and for the number of branches per plant (34.39% and 23.55%, respectively). For the earliness traits, negative heterosis is considered desirable, and 11 hybrids exhibited significant negative hb for days to first flowering. Among them, SL-8-PB-1-3-1-4 × Pusa Purple Round showed the highest negative hb (−17.58%) and hs (−17.08%), followed by BL-2011-219-8-1 × Pusa Purple Long. Similar trends were observed for days to 50% flowering and days to first fruit harvest, confirming the superiority of these hybrids for earliness. Among the reproductive traits, BL-2011-219-8-1 × SL-8-PB-1-3-1–4 exhibited the highest significant positive hb for number of flowers per cluster (21.78%), number of fruits per cluster (37.31%), fruit set percentage (32.38%), and number of fruits per plant (38.35%) while also expressing high hs over ‘Kalpataru’ for these traits. For the fruit characteristics, Pant Rituraj × S-324-465-2–2 recorded the highest hb for fruit length (18.91%) and average fruit weight (49.03%), whereas Pusa Purple Round × Pant Rituraj exhibited the highest heterosis for fruit diameter over both the better parent and standard check. Significant positive heterosis for total fruit yield per plant was observed in several hybrids, with SL-8-PB-1-3-1-4 × Pant Rituraj showing the highest hb (56.93%), while Pusa Purple Long × BL-219 recorded the highest hs (39.11%) over ‘Kalpataru’. For the quality traits, Pant Rituraj × S-324-465-2–2 exhibited the highest hb for TSS (37.36%) and anthocyanin content (144.63%), whereas Pusa Purple Long × BL-219 recorded the highest hs for anthocyanin content (20.59%) over the commercial check ‘Kalpataru’.

### 3.6 Identification of top hybrids based on SCA, per se performance, heterobeltiosis, and standard heterosis

Enhancing fruit yield is a primary objective of crop improvement programs. Based on the integrated evaluation presented in **Table 4**, several cross combinations were repeatedly identified across SCA effects, per se performance, hb, and hs for important growth, flowering, yield, and quality traits, indicating their strong breeding potential and commercial significance. Among the evaluated hybrids, BL-2011-219-8-1 × SL-8-PB-1-3-1–4 emerged as one of the most consistently superior combinations as it was repeatedly identified for plant height, number of branches, flowering and fruiting traits, fruit set percentage, number of fruits per plant, fruit length, fruit yield per plant, and several quality attributes. The repeated superiority of this hybrid across multiple selection criteria suggests strong parental complementation and favorable gene interactions contributing to enhanced hybrid vigor, reproductive efficiency, and stable trait expression. Similarly, Pusa Purple Long × BL-219 exhibited superior performance for branching, reproductive traits, average fruit weight, fruit yield, TSS, iron content, and anthocyanin content, indicating the importance of non-additive gene action and its suitability for commercial hybrid development. Pant Rituraj × S-324-465-2–2 also showed repeated superiority for fruit size, fruit weight, yield, and quality traits, particularly the TSS and anthocyanin contents, suggesting its usefulness for simultaneous improvement of productivity and nutritional quality. For earliness and reduced acidity, SL-8-PB-1-3-1-4 × Pusa Purple Round and BL-2011-219-8-1 × Pusa Purple Long were repeatedly identified across SCA, hb, and hs estimates, indicating their potential for the development of early maturing hybrids with desirable fruit quality.

**Table 4:** Representation of the top hybrids based on specific combining ability (SCA), per se performance, heterobeltiosis, and standard heterosis.

Trait	Specific combiners	Per se performance	Heterobeltiosis	Standard heterosis
PH	Pusa Purple Long × BL-219 BL-2011-219-8-1 × Pusa Purple Long BL-2011-219-8-1 × SL-8-PB-1-3-1-4	BL-2011-219-8-1 × SL-8-PB-1-3-1-4 BL-2011-219-8-1 × Pusa Purple Long SL-8-PB-1-3-1-4 × Pusa Purple Long	BL-2011-219-8-1 × SL-8-PB-1-3-1-4 SL-8-PB-1-3-1-4 × Pusa Purple Long SL-8-PB-1-3-1-4 × Pant Rituraj	BL-2011-219-8-1 × SL-8-PB-1-3-1-4 BL-2011-219-8-1 × Pusa Purple Long SL-8-PB-1-3-1-4 × Pusa Purple Long
NB	Pusa Purple Long × BL-219 BL-2011-219-8-1 × SL-8-PB-1-3-1-4 SL-8-PB-1-3-1-4 × Pusa Purple Long	BL-2011-219-8-1 × SL-8-PB-1-3-1-4 SL-8-PB-1-3-1-4 × Pusa Purple Long Pusa Purple Long × BL-219	BL-2011-219-8-1 × SL-8-PB-1-3-1-4 SL-8-PB-1-3-1-4 × Pusa Purple Long Pusa Purple Long × BL-219	BL-2011-219-8-1 × SL-8-PB-1-3-1-4 SL-8-PB-1-3-1-4 × Pusa Purple Long Pusa Purple Long × BL-219
DFF	SL-8-PB-1-3-1-4 × Pusa Purple Round BL-219 × Pusa Purple Round BL-2011-219-8-1 × Pusa Purple Long	BL-2011-219-8-1 × Pusa Purple Long BL-2011-219-8-1 × SL-8-PB-1-3-1-4 SL-8-PB-1-3-1-4 × Pusa Purple Long	SL-8-PB-1-3-1-4 × Pusa Purple Round BL-2011-219-8-1 × Pusa Purple Long BL-219 × Pusa Purple Round	SL-8-PB-1-3-1-4 × Pusa Purple Round BL-2011-219-8-1 × Pusa Purple Long BL-219 × Pusa Purple Round
D50%F	SL-8-PB-1-3-1-4 × Pusa Purple Round BL-219 × Pusa Purple Round BL-2011-219-8-1 × Pusa Purple Long	BL-2011-219-8-1 × Pusa Purple Long BL-2011-219-8-1 × SL-8-PB-1-3-1-4 SL-8-PB-1-3-1-4 × Pusa Purple Long	SL-8-PB-1-3-1-4 × Pusa Purple Round BL-2011-219-8-1 × Pusa Purple Long BL-219 × Pusa Purple Round	SL-8-PB-1-3-1-4 × Pusa Purple Round BL-2011-219-8-1 × Pusa Purple Long BL-219 × Pusa Purple Round
NFLC	Pusa Purple Long × BL-219 BL-2011-219-8-1 × SL-8-PB-1-3-1-4 Pant Rituraj × S-324-465-2-2	BL-2011-219-8-1 × SL-8-PB-1-3-1-4 SL-8-PB-1-3-1-4 × Pusa Purple Long BL-2011-219-8-1 × Pusa Purple Long Pusa Purple Long × BL-219	BL-2011-219-8-1 × SL-8-PB-1-3-1-4 SL-8-PB-1-3-1-4 × Pusa Purple Long Pusa Purple Long × BL-219	BL-2011-219-8-1 × SL-8-PB-1-3-1-4 SL-8-PB-1-3-1-4 × Pusa Purple Long Pusa Purple Long × BL-219
NFC	Pusa Purple Long × BL-219 BL-2011-219-8-1 × SL-8-PB-1-3-1-4 SL-8-PB-1-3-1-4 × Pusa Purple Long	BL-2011-219-8-1 × SL-8-PB-1-3-1-4 SL-8-PB-1-3-1-4 × Pusa Purple Long BL-2011-219-8-1 × Pusa Purple Long Pusa Purple Long × BL-219	BL-2011-219-8-1 × SL-8-PB-1-3-1-4 SL-8-PB-1-3-1-4 × Pusa Purple Long Pusa Purple Long × BL-219	BL-2011-219-8-1 × SL-8-PB-1-3-1-4 SL-8-PB-1-3-1-4 × Pusa Purple Long Pusa Purple Long × BL-219
FS	BL-2011-219-8-1 × Pusa Purple Long BL-2011-219-8-1 × SL-8-PB-1-3-1-4 Pusa Purple Long × BL-219	BL-2011-219-8-1 × SL-8-PB-1-3-1-4 BL-2011-219-8-1 × Pusa Purple Long SL-8-PB-1-3-1-4 × Pusa Purple Long	BL-2011-219-8-1 × SL-8-PB-1-3-1-4 SL-8-PB-1-3-1-4 × Pusa Purple Long Pusa Purple Long × BL-219	BL-2011-219-8-1 × SL-8-PB-1-3-1-4 SL-8-PB-1-3-1-4 × Pusa Purple Long Pusa Purple Long × BL-219
DFH	SL-8-PB-1-3-1-4 × Pusa Purple Round BL-219 × Pusa Purple Round BL-2011-219-8-1 × Pusa Purple Long	BL-2011-219-8-1 × Pusa Purple Long SL-8-PB-1-3-1-4 × Pusa Purple Round BL-2011-219-8-1 × SL-8-PB-1-3-1-4	SL-8-PB-1-3-1-4 × Pusa Purple Round BL-2011-219-8-1 × Pusa Purple Long BL-219 × Pusa Purple Round	SL-8-PB-1-3-1-4 × Pusa Purple Round BL-2011-219-8-1 × Pusa Purple Long BL-219 × Pusa Purple Round
NFPP	Pusa Purple Long × BL-219 BL-2011-219-8-1 × SL-8-PB-1-3-1-4 SL-8-PB-1-3-1-4 × Pusa Purple Long	BL-2011-219-8-1 × SL-8-PB-1-3-1-4 SL-8-PB-1-3-1-4 × Pusa Purple Long Pusa Purple Long × BL-219	BL-2011-219-8-1 × SL-8-PB-1-3-1-4 SL-8-PB-1-3-1-4 × Pusa Purple Long Pusa Purple Long × BL-219	BL-2011-219-8-1 × SL-8-PB-1-3-1-4 SL-8-PB-1-3-1-4 × Pusa Purple Long Pusa Purple Long × BL-219
FL	BL-2011-219-8-1 × SL-8-PB-1-3-1-4 Pusa Purple Long × BL-219 Pant Rituraj × S-324-465-2-2	BL-2011-219-8-1 × SL-8-PB-1-3-1-4 BL-2011-219-8-1 × Pusa Purple Long SL-8-PB-1-3-1-4 × Pusa Purple Long Pusa Purple Long × BL-219	BL-2011-219-8-1 × SL-8-PB-1-3-1-4 Pant Rituraj × S-324-465-2-2 SL-8-PB-1-3-1-4 × Pant Rituraj	BL-2011-219-8-1 × SL-8-PB-1-3-1-4 SL-8-PB-1-3-1-4 × Pant Rituraj Pant Rituraj × S-324-465-2-2
FD	Pusa Purple Round × Pant Rituraj BL-219 × BLW-2001-1-1-2 Pant Rituraj × S-324-465-2-2	BL-219 × BLW-2001-1-1-2 Pusa Purple Round × Pant Rituraj Pusa Purple Round × BLW-2001-1-1-2	BL-219 × BLW-2001-1-1-2 Pusa Purple Round × Pant Rituraj Pant Rituraj × S-324-465-2-2	BL-219 × BLW-2001-1-1-2 Pusa Purple Round × Pant Rituraj Pant Rituraj × S-324-465-2-2
FWT	Pusa Purple Long × BL-219 Pant Rituraj × S-324-465-2-2 Pusa Purple Round × Pant Rituraj	Pusa Purple Long × BL-219 Pant Rituraj × S-324-465-2-2 Pusa Purple Round × Pant Rituraj	Pant Rituraj × S-324-465-2-2 SL-8-PB-1-3-1-4 × Pant Rituraj Pusa Purple Long × BL-219	Pant Rituraj × S-324-465-2-2 Pusa Purple Round × Pant Rituraj Pusa Purple Round × BLW-2001-1-1-2
FYPP	Pusa Purple Long × BL-219 Pant Rituraj × S-324-465-2-2 SL-8-PB-1-3-1-4 × Pant Rituraj	Pusa Purple Long × BL-219 BL-2011-219-8-1 × SL-8-PB-1-3-1-4 Pant Rituraj × S-324-465-2-2	SL-8-PB-1-3-1-4 × Pant Rituraj Pant Rituraj × S-324-465-2-2 Pusa Purple Long × BL-219	Pusa Purple Long × BL-219 BL-2011-219-8-1 × SL-8-PB-1-3-1-4 Pant Rituraj × S-324-465-2-2
FE	Pusa Purple Long × BL-219 SL-8-PB-1-3-1-4 × Pusa Purple Long Pant Rituraj × S-324-465-2-2	Pusa Purple Long × BL-219 SL-8-PB-1-3-1-4 × Pusa Purple Long SL-8-PB-1-3-1-4 × Pusa Purple Round	SL-8-PB-1-3-1-4 × Pusa Purple Long Pusa Purple Long × BL-219 BL-2011-219-8-1 × SL-8-PB-1-3-1-4	Pusa Purple Long × BL-219 SL-8-PB-1-3-1-4 × Pusa Purple Long BL-2011-219-8-1 × SL-8-PB-1-3-1-4
TSS	Pusa Purple Long × BL-219 SL-8-PB-1-3-1-4 × Pusa Purple Long Pant Rituraj × S-324-465-2-2	Pusa Purple Long × BL-219 SL-8-PB-1-3-1-4 × Pusa Purple Long SL-8-PB-1-3-1-4 × Pusa Purple Round	Pant Rituraj × S-324-465-2-2 BL-2011-219-8-1 × SL-8-PB-1-3-1-4 SL-8-PB-1-3-1-4 × Pant Rituraj	Pusa Purple Long × BL-219 SL-8-PB-1-3-1-4 × Pusa Purple Round BL-2011-219-8-1 × SL-8-PB-1-3-1-4
ACD	SL-8-PB-1-3-1-4 × Pusa Purple Round BL-219 × Pusa Purple Round BL-2011-219-8-1 × Pusa Purple Long	SL-8-PB-1-3-1-4 × Pusa Purple Round BL-2011-219-8-1 × Pusa Purple Long BL-2011-219-8-1 × SL-8-PB-1-3-1-4	SL-8-PB-1-3-1-4 × Pusa Purple Round BL-2011-219-8-1 × Pusa Purple Long BL-219 × Pusa Purple Round	SL-8-PB-1-3-1-4 × Pusa Purple Round BL-2011-219-8-1 × Pusa Purple Long BL-219 × Pusa Purple Round
ANTH	Pusa Purple Long × BL-219 SL-8-PB-1-3-1-4 × Pusa Purple Long Pant Rituraj × S-324-465-2-2	Pusa Purple Long × BL-219 SL-8-PB-1-3-1-4 × Pusa Purple Long SL-8-PB-1-3-1-4 × Pusa Purple Round	Pant Rituraj × S-324-465-2-2 Pusa Purple Round × Pant Rituraj SL-8-PB-1-3-1-4 × Pant Rituraj	Pusa Purple Long × BL-219 SL-8-PB-1-3-1-4 × Pusa Purple Long SL-8-PB-1-3-1-4 × Pusa Purple Round

PH, plant height; NB, number of branches per plant; DFF, days to first flowering; D50%F, days to 50% flowering; NFLC, number of flowers per flower cluster; NFC, number of fruits per cluster; FS, fruit set (in percent); DFH, days to first harvest; NFPP, number of fruits per plant; FL, fruit length; FD, fruit diameter; FWT, fruit weight; FYPP, fruit yield per plant; FE, iron; TSS, total soluble solids; ACD, acidity; ANTH, anthocyanin content.

### 3.7 Genotype × environment interaction

The stability analysis revealed significant G × E interactions for the growth, flowering, maturity, yield, and quality traits among parents and hybrids, indicating differential environmental responsiveness and adaptability (**Supplementary Table 4**). Plant height ranged from 45.63 cm (Pant Rituraj) to 56.87 cm (Pusa Purple Round) among parents, with a regression coefficient (*b_i_*) in the range 0.70–1.12. A non-significant deviation from regression (*S*^2^*d_i_* ≈ 0) was observed in the majority of the parents, suggesting a predictable performance across environments. The plant height of hybrids ranged from 45.73 cm (BL-219 × BLW-2001-1-1-2) to 73.99 cm (BL-2011-219-8-1 × SL-8-PB-1-3-1-4), and the *b_i_* values ranged 0.16–1.16, indicating differences in the response to environment. Similar variability was observed for number of branches at 90 DAT, flowering, and the reproductive and maturity traits. The days to first flowering among hybrids varied from 57.76 days in SL-8-PB-1-3-1-4 × Pusa Purple Round to 72.35 days in BL-219 × BLW-2001-1-1-2. Days to 50% flowering and days to first fruit harvest also varied considerably in different environments. Among the reproductive traits, BL-2011-219-8-1 × SL-8-PB-1-3-1–4 recorded the maximum number of flowers per cluster (6.23), fruits per cluster (4.93), and fruit set percentage (79.17%), with *b_i_* < 1 and non-significant *S*^2^*d_i_* indicating stable performance with wide adaptability. The yield and yield contributing traits also followed a high degree of variability, with the fruit yield per plant ranging from 671.43 to 1,315.24 g among hybrids, with the maximum yield recorded by Pusa Purple Long × BL-219 (total fruit yield of 548.02 q ha^−1^). Similarly, the fruit length, fruit diameter, number of fruits per plant, and mean fruit weight exhibited differential adaptability among the environments. The quality traits also showed significant variations, with TSS ranging from 5.33 to 6.95°Brix, titratable acidity from 0.24 to 0.33, and anthocyanin content from 14.60 to 22.61 mg 100 g^−1^ among hybrids, indicating the influence of environmental conditions on the fruit quality parameters.

**Figure 4:**
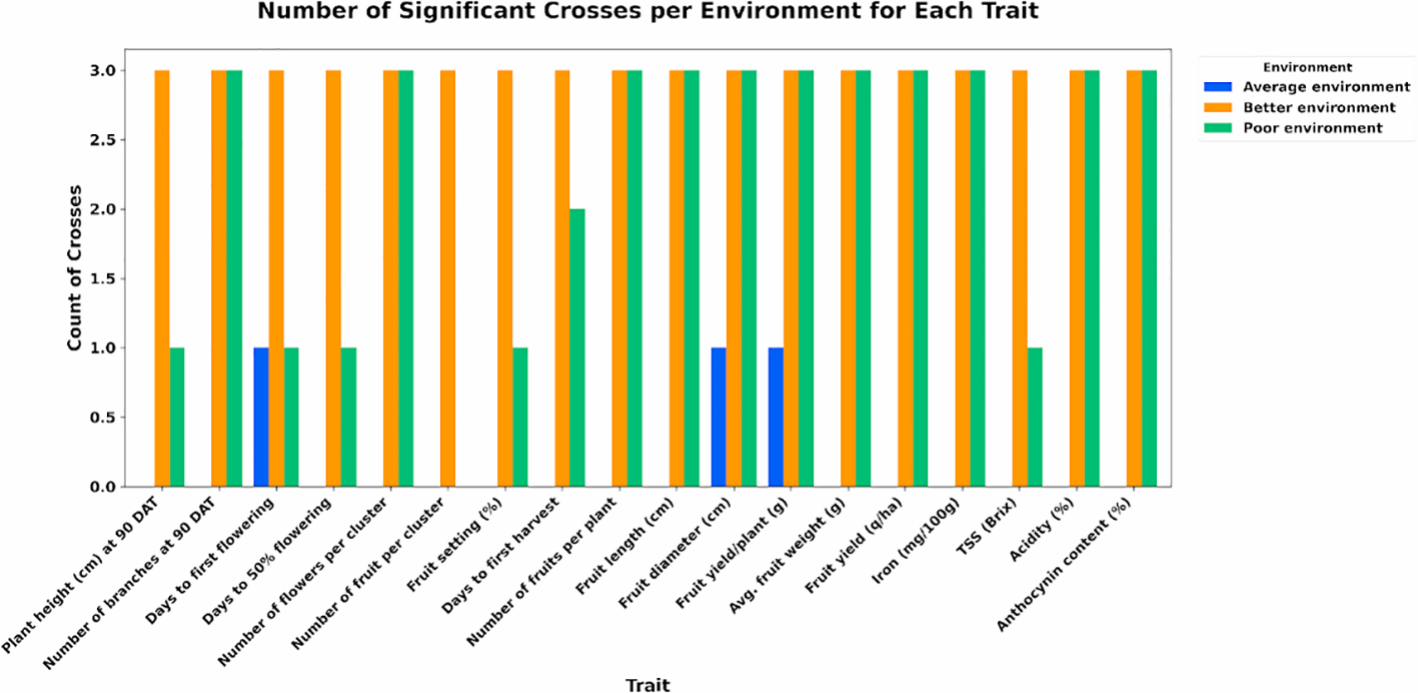
Number of significant crosses identified for different yield and quality traits under average, better, and poor environments.

Based on the regression coefficient (*b_i_*) and deviation from regression (*S*^2^*d_i_*), the stable hybrids were categorized into favorable, poor, and average adaptability groups (**Supplementary Table 5**; **Figure 4**). Hybrids with *b_i_* > 1 and non-significant *S*^2^*d_i_* were considered suitable for favorable environments, whereas those with *b_i_* < 1 and non-significant *S*^2^*d_i_* were considered better adapted to poor environments. Genotypes with *b_i_* ≈ 1 and *S*^2^*d_i_* ≈ 0 were regarded as stable across environments with wide adaptability. Accordingly, Pusa Purple Long × BL-219, SL-8-PB-1-3-1-4 × Pusa Purple Long, and BL-2011-219-8-1 × SL-8-PB-1-3-1–4 were identified as superior hybrids for favorable environments for the major yield and quality traits. In contrast, BL-2011-219-8-1 × Pusa Purple Long and BL-2011-219-8-1 × Pusa Purple Round exhibited comparatively better adaptation under poor environments for specific traits. Furthermore, BL-2011-219-8-1 × SL-8-PB-1-3-1–4 showed *b_i_* values close to unity with negligible deviation from regression, indicating average stability and wide adaptability across environments. The results confirmed the existence of specific and broad adaptability of the examined hybrids and showed their possible usefulness for specific cultivation in various agro-ecological conditions.

## 4 Discussion

Developing high-yielding brinjal cultivars needs the fine selection of suitable parental lines and superior F_1_ hybrids, which remains a critical hurdle for plant breeders. Genetic enhancement mainly involves the screening of parents with desirable characteristics and the combinations of crosses capable of improving yield and its component characteristics. Heterosis breeding has been widely exploited in brinjal because its fruit yield and related traits are quantitatively inherited and strongly influenced by both additive and non-additive genetic effects. In the present study, significant variations among the parents and hybrids for the growth, flowering, yield, and quality traits demonstrated the existence of substantial genetic variability, thereby providing adequate scope for effective selection and hybrid improvement. Combining ability analysis further helped in understanding the relative importance of the additive and dominance gene effects governing these traits.

The significant heterosis observed for fruit yield and its component characteristics indicated the presence of favorable genetic interactions among parental lines. The superiority of certain hybrids for the yield and quality traits may be attributed to dominance, overdominance, and complementary epistatic interactions, which collectively enhanced vegetative vigor, assimilate partitioning efficiency, and reproductive performance. Enhanced source–sink relationships, better translocation of photosynthates toward developing fruits, and improved fruit-setting efficiency may also have contributed to the higher hybrid productivity. The earliness observed in some hybrids through significant negative heterosis for the flowering and harvesting traits may be associated with the faster floral initiation and efficient utilization of assimilates during the early growth stages. Similar findings for heterosis in brinjal have also been reported by Gondaliya et al. (2024); Phor et al. (2022); Tripathy et al. (2025); Mahla et al. (2025); Reddy et al. (2020), and Patel et al. (2017).

The combining ability analysis revealed the importance of both additive and non-additive gene actions in the inheritance of the studied characteristics. Parents exhibiting high GCA effects, particularly SL-8-PB-1-3-1–4 and BL-2011-219-8-1, indicated the presence of favorable additive gene effects and their ability to transmit desirable alleles consistently to progenies. At the same time, the superior SCA effects observed in hybrids such as Pusa Purple Long × BL-219 and BL-2011-219-8-1 × SL-8-PB-1-3-1–4 indicated the importance of non-additive genetic interactions, particularly dominance effects, in the expression of yield and quality traits Although several traits exhibited appreciable GCA effects, the predictability ratio below unity along with the higher contribution of SCA variance suggested a relatively greater role of non-additive gene action in governing these characteristics. These findings indicate that exploitation of heterosis through hybrid breeding would be more effective for improving fruit yield and associated traits in brinjal. Similar observations were earlier reported by Mishra et al. (2023); Shrivastav et al. (2024); Anvesh et al. (2024); Keerthana et al. (2025), and Dudhatra et al. (2024).

The significant G × E interactions observed for all major traits indicated differential responses of the genotypes under the tested environments, emphasizing the importance of multi-environment evaluation before varietal recommendation. As the environments in the present investigation were generated under different seasonal conditions at the same experimental location, the observed stability primarily reflects adaptability across the tested seasonal environments rather than across diverse agro-ecological zones. Genotypes exhibiting regression coefficient (*b_i_*) values close to unity with non-significant deviation from regression (*S*^2^*d_i_* ≈ 0) demonstrated predictable and stable performance. Hybrids such as BL-2011-219-8-1 × SL-8-PB-1-3-1–4 and Pusa Purple Long × BL-219 combined superior yield performance with desirable stability parameters, indicating their potential suitability for cultivation under similar production environments. The presence of significant G × E interactions further suggested that environmental factors influenced the magnitude of hybrid expression for the yield and quality traits, which highlights the necessity for continued testing across seasons and management conditions. Similar observations were made by Kumawat et al. (2023); Akhtar et al. (2019); Vaddoria et al. (2009), and Lila et al. (2011). Some hybrids displayed high heterotic responses, which might be due to the poor performance of parents but good complementation between genetically diverse parents resulting in higher hybrid vigor. The simultaneous consideration of parental performance and hybrid superiority, and not percentage values for the interpretation of heterosis, is necessary.

## 5 Conclusion

The present investigation revealed wide variability among the parents and hybrids for the growth, flowering, yield, and quality traits in brinjal. The inheritance of the studied characteristics was governed by both additive and non-additive gene actions, but the predominance of SCA variance and predictability ratios less than unity indicated the relatively greater role of non-additive gene action for the majority of the yield and quality traits. The parents BL-2011-219-8–1 and SL-8-PB-1-3-1–4 were identified as good general combiners for several economically important characteristics. Hybrids such as Pusa Purple Long × BL-219, Pant Rituraj × S-324-465-2-2, and BL-2011-219-8-1 × SL-8-PB-1-3-1–4 showed excellent per se performance, high heterosis, and desirable SCA effects for fruit yield and related traits. Further stability analysis revealed the significant and positive environment effects, with the cross BL-2011-219-8-1 × SL-8-PB-1-3-1–4 exhibiting relatively stable performance and desirable adaptability parameters. These results underline the utility of combining ability, heterosis, and stability analyses in the selection of suitable hybrids for future brinjal breeding programs and commercial hybrid development after further screening at multiple locations and for consumer preference.

## Data Availability

The original contributions presented in the study are included in the article/**Supplementary Material**. Further inquiries can be directed to the corresponding authors.
