# Supplementary material for: Heterotic potential, combining ability, and stability analysis for yield and quality attributes in eggplant (Solanum melanogena)
Source: Front Plant Sci. 2026 Jun 30;17:1836360. doi: 10.3389/fpls.2026.1836360 (PMC13364897; doi:10.3389/fpls.2026.1836360)
Supplement: Supplementary file 1 [file Table1.docx]

**Supplementary Table 1.** Mean performance of general combining ability effects of parents in growth, yield and quality traits in brinjal. The symbols * and ** represent significance at P < 0.05 and P < 0.01 levels, respectively.

| **Parents** | **Plant height**  **(cm) at 90 DAT** | **Number of branches**  **at 90 DAT** | **Days to first flowering** | **Days to 50% flowering** | **Number of flowers per cluster** | **number of fruits per cluster** | **Fruit setting (%)** | **Days to first fruit harvest** | **Number of fruits per plant** | **Fruit length**  **(cm)** | **Fruit diameter**  **(cm^2^)** | **fruit yield plant^-1^ (g)** | **Average fruit weight (g)** | **Fruit yield**  **(q ha^-1^)** | **Iron (mg 100g^-1^)** | **Total soluble solid (^o^B)** | **Acidity (%)** | **Anthocyanin (%)** |
| --- | --- | --- | --- | --- | --- | --- | --- | --- | --- | --- | --- | --- | --- | --- | --- | --- | --- | --- |
| **BL-2011-219-8-1** | 3.034 ** | 0.375 ** | -0.659 ** | -0.688 ** | 0.167 ** | 0.199 ** | 2.947 ** | -0.743 ** | 0.551 ** | 0.682 ** | -0.187 ** | 57.125 ** | 1.725 ** | 23.802 ** | 0.007 ** | 0.129 ** | -0.003 | 0.640 ** |
| **SL-8-PB-1-3-1-4** | 3.252 ** | 0.437 ** | -3.092 ** | -3.250 ** | 0.195 ** | 0.233 ** | 3.158 ** | -3.499 ** | 0.655 ** | 0.647 ** | -0.699 ** | 3.331 | -4.092 ** | 1.388 | 0.009 ** | 0.171 ** | -0.017 ** | 0.870 ** |
| **Pusa Purple Long** | 0.735 | 0.151 * | -1.342 ** | -1.445 ** | 0.067 * | 0.075 * | 0.422 | -1.564 ** | 0.206 * | 0.542 ** | -0.341 ** | 12.58 | -0.481 | 5.242 | 0.003 | 0.049 | -0.007 ** | 0.261 |
| **BL-219** | -1.648 ** | -0.179 ** | 1.275 ** | 1.349 ** | -0.080 ** | -0.094 ** | -0.666 | 1.471 ** | -0.254 ** | -0.346 ** | 0.318 ** | -1.567 | 1.351 * | -0.653 | -0.003 | -0.054 | 0.007 ** | -0.243 |
| **Pusa Purple Round** | 0.558 | 0.038 | 1.129 ** | 1.194 ** | 0.017 | 0.02 | 0.231 | 1.281 ** | 0.023 | -0.14 | 0.289 ** | 21.444 * | 2.317 ** | 8.935 * | 0.002 | 0.031 | 0.005 ** | 0.138 |
| **Pant Rituraj** | -2.755 ** | -0.369 ** | -0.11 | -0.098 | -0.166 *** | -0.198 ** | -3.348 ** | -0.114 | -0.515** | -0.543 ** | 0.031 | -53.024 ** | -2.203 ** | -22.093 ** | -0.006 ** | -0.117 ** | 0 | -0.625 ** |
| **S-324-465-2-2** | -1.326 ** | -0.196 ** | 1.100 ** | 1.154 ** | -0.087 ** | -0.100 ** | -1.815 ** | 1.253 ** | -0.299 ** | -0.236 * | 0.181 ** | -14.72 | 0.875 | -6.133 | -0.006 * | -0.103 * | 0.006 ** | -0.508 * |
| **BLW-2001-1-1-2** | -1.849 ** | -0.258 ** | 1.699 ** | 1.784 ** | -0.113 *** | -0.135 ** | -0.93 | 1.915 ** | -0.368** | -0.606 ** | 0.407 ** | -25.167 ** | 0.508 | -10.486 ** | -0.006 * | -0.105 * | 0.009 ** | -0.533 ** |
| **Gi-Gj 95%** | 1.488 | 0.186 | 0.692 | 0.729 | 0.083 | 0.101 | 1.624 | 0.784 | 0.282 | 0.287 | 0.146 | 26.837 | 1.978 | 11.182 | 0.007 | 0.124 | 0.005 | 0.61 |
| **Gi-Gj 99%** | 1.962 | 0.245 | 0.912 | 0.961 | 0.109 | 0.134 | 2.142 | 1.034 | 0.372 | 0.378 | 0.192 | 35.388 | 2.608 | 14.745 | 0.009 | 0.163 | 0.006 | 0.804 |

**Supplementary Table 2.** Mean performance of specific combining ability effects of parents in growth, yield and quality traits in brinjal. The symbols * and ** represent significance at P < 0.05 and P < 0.01 levels, respectively.

| **Hybrid** | **Plant height**  **(cm) at 90 DAT** | **Number of branches**  **at 90 DAT** | **Days to first flowering** | **Days to 50% flowering** | **Number of flowers per cluster** | **Number of fruits per cluster** | **Fruit setting (%)** | **Days to first fruit harvest** | **Number of fruits per plant** | **Fruit length**  **(cm)** | **Fruit diameter**  **(cm^2^)** | **Fruit yield plant^-1^ (g)** | **Average fruit weight (g)** | **Fruit yield**  **(q ha^-1^)** | **Iron**  **(mg 100g^-1^)** | **Total soluble solid (^o^B)** | **Acidity (%)** | **Anthocyanin (%)** |
| --- | --- | --- | --- | --- | --- | --- | --- | --- | --- | --- | --- | --- | --- | --- | --- | --- | --- | --- |
| **BL-2011-219-8-1 x SL-8-PB-1-3-1-4** | 9.25 ** | 1.39 ** | -2.59** | -2.74 ** | 0.62 ** | 0.74 ** | 10.47** | -2.95 ** | 2.30 ** | 2.16 ** | -0.50 ** | 227.33 ** | 3.77 | 94.72 ** | 0.015 * | 0.300 * | -0.012 * | 1.476 * |
| **BL-2011-219-8-1 x Pusa Purple Long** | 9.62 ** | 1.08 ** | -5.05 ** | -5.29 ** | 0.48 ** | 0.58 ** | 10.78 ** | -5.69 ** | 1.66 ** | 1.28 ** | -1.01 ** | 42.57 | -6.29 ** | 17.74 | 0.024 ** | 0.458 ** | -0.026 ** | 2.275 ** |
| **BL-2011-219-8-1 x BL-219** | -1.41 | -0.24 | -0.08 | -0.09 | -0.10 | -0.12 | -2.61 | -0.12 | -0.36 | -0.44 | -0.07 | -60.56 * | -2.67 | -25.23 * | -0.004 | -0.079 | -0.001 | -0.42 |
| **BL-2011-219-8-1 x Pusa Purple Round** | 1.18 | 0.137 | 1.72 * | 1.81 * | 0.05 | 0.06 | 1.59 | 1.96 * | 0.21 | -0.29 | 0.31 * | 48.59 | 2.21 | 20.25 | 0.01 | 0.2 | 0.010 * | 1.015 |
| **BL-2011-219-8-1 x Pant Rituraj** | 1.42 | 0.17 | 2.76 ** | 2.89 ** | 0.08 | 0.09 | 1.83 | 3.12 ** | 0.21 | 0.07 | 0.52 ** | 75.44 ** | 6.23 ** | 31.43 ** | 0.006 | 0.079 | 0.014 ** | 0.438 |
| **BL-2011-219-8-1 x S-324-465-2-2** | -1.10 | -0.14 | 0.57 | 0.61 | -0.06 | -0.07 | -0.89 | 0.64 | -0.21 | -0.45 | 0.17 | -19.15 | -0.49 | -7.98 | 0.001 | 0.007 | 0.003 | 0.029 |
| **BL-2011-219-8-1 x BLW-2001-1-1-2** | -2.46 | -0.31 | -1.56 * | -1.63 * | -0.14 | -0.16 | -3.82 * | -1.76 * | -0.47 | -0.40 | -0.38 * | -93.51 ** | -5.48 ** | -38.96 ** | -0.007 | -0.122 | -0.008 | -0.602 |
| **SL-8-PB-1-3-1-4 x Pusa Purple Long** | 7.78 ** | 1.15** | -1.79 * | -1.85 * | 0.51 ** | 0.62 ** | 8.79 ** | -2.01 * | 1.67 ** | 1.32 ** | -0.33 * | 143.42 ** | 2.13 | 59.76 ** | 0.033 ** | 0.625 ** | -0.008 | 2.999 ** |
| **SL-8-PB-1-3-1-4 x BL-219** | -0.49 | -0.16 | 0.15 | 0.15 | -0.07 | -0.08 | -1.69 | 0.24 | -0.26 | -0.19 | -0.02 | -37.86 | -0.98 | -15.78 | -0.002 | -0.035 | 0.002 | -0.221 |
| **SL-8-PB-1-3-1-4 x Pusa Purple Round** | 7.16 ** | 0.84 ** | -5.64 ** | -5.94 ** | 0.37 ** | 0.45 ** | 8.12 ** | -6.41 ** | 1.22 ** | 0.58 * | -1.24 ** | -54.80 * | -11.19 ** | -22.83 * | 0.028 ** | 0.524 ** | -0.042 ** | 2.601 ** |
| **SL-8-PB-1-3-1-4 x Pant Rituraj** | 5.69 ** | 0.66 ** | 2.44 ** | 2.55 ** | 0.29 ** | 0.35 ** | 6.51 ** | 2.74 ** | 0.91 ** | 0.91 ** | 0.45 ** | 183.69 ** | 10.39 ** | 76.53 ** | 0.022 ** | 0.407 ** | 0.014 ** | 1.936 ** |
| **SL-8-PB-1-3-1-4 x S-324-465-2-2** | -2.58 | -0.36 | 1.99 ** | 2.11 ** | -0.16 | -0.19 | -2.47 | 2.25 ** | -0.53 | -0.63 * | 0.47 ** | -25.64 | 1.59 | -10.68 | -0.006 | -0.119 | 0.012 * | -0.622 |
| **SL-8-PB-1-3-1-4 x BLW-2001-1-1-2** | 3 | 0.32 | 0.94 | 0.99 | 0.14 | 0.17 | 2.03 | 1.07 | 0.44 | -0.35 | 0.14 | 35.12 | 0.37 | 14.63 | 0.014 * | 0.241 | 0.006 | 1.192 |
| **Pusa Purple Long x BL-219** | 9.69 ** | 1.62 ** | 3.37 ** | 3.57 ** | 0.72 ** | 0.86 ** | 9.38 ** | 3.83 ** | 2.44** | 1.62 ** | 0.64 ** | 437.64 ** | 18.78 ** | 182.34 ** | 0.054 ** | 1.010 ** | 0.017 ** | 4.992 ** |
| **Pusa Purple Long x Pusa Purple Round** | -1.47 | -0.22 | 0.33 | 0.25 | -0.09 | -0.13 | -1.28 | 0.21 | -0.48 | -0.69 * | -0.003 | -59.75 * | -2.81 | -24.89 * | -0.004 | -0.084 | 0.002 | -0.336 |
| **Pusa Purple Long x Pant Rituraj** | -0.09 | -0.06 | 0.56 | 0.61 | -0.04 | -0.07 | 0.20 | 0.61 | -0.13 | -0.37 | 0.07 | -33.29 | -1.27 | -13.87 | -0.005 | -0.083 | 0.002 | -0.412 |
| **Pusa Purple Long x S-324-465-2-2** | -5.59** | -0.75 ** | 0.66 | 0.74 | -0.33** | -0.39 ** | -5.74 ** | 0.78 | -1.07 ** | -0.51 | 0.19 | -95.83 ** | -0.93 | -39.93 ** | -0.022 ** | -0.416 ** | 0.003 | -1.992 ** |
| **Pusa Purple Long x BLW-2001-1-1-2** | -7.14 ** | -0.93 ** | -1.69 * | -1.74 * | -0.41 ** | -0.50 ** | -8.89 ** | -1.79 * | -1.37 ** | -0.51 | -0.40 ** | -169.66 ** | -6.35 ** | -70.69 ** | -0.030 ** | -0.563 ** | -0.009 | -2.724 ** |
| **BL-219 x Pusa Purple Round** | -3.39 * | -0.45 * | -5.28 ** | -5.56 ** | -0.19 * | -0.23 * | -4.85 ** | -5.92 ** | -0.63 * | -0.42 | -1.27 ** | -215.03 ** | -15.59 ** | -89.59 ** | -0.017 * | -0.319 * | -0.026 ** | -1.605 * |
| **BL-219 x Pant Rituraj** | 2.71 | 0.31 | -1.16 | -1.24 | 0.13 | 0.15 | 1.97 | -1.38 | 0.52 | 0.59 * | -0.40 ** | 23.04 | -1.19 | 9.60 | 0.002 | 0.045 | -0.006 | 0.005 |
| **BL-219 x S-324-465-2-2** | 1.39 | 0.14 | -3.06 ** | -3.22 ** | 0.07 | 0.08 | 0.35 | -3.41 ** | 0.15 | 0.14 | -0.69 ** | -67.22 * | -6.67 ** | -28.01 * | 0.002 | 0.036 | -0.016 ** | 0.03 |
| **BL-219 x BLW-2001-1-1-2** | -9.22 ** | -1.15 ** | 4.01 ** | 4.23 ** | -0.51 ** | -0.62 ** | 2.01 | 4.52 ** | -1.71 ** | -1.44 ** | 1.69 ** | -100.00 ** | 4.72 * | -41.67 ** | -0.023 ** | -0.425 ** | 0.020 ** | -2.169 ** |
| **Pusa Purple Round x Pant Rituraj** | -3.04 * | -0.35 | 4.98 ** | 5.35 ** | -0.15 | -0.18 | -3.00 | 5.76 ** | -0.50 | -0.36 | 1.91 ** | 113.26 ** | 15.97 ** | 47.19 ** | -0.017 * | -0.314 * | 0.027 ** | -1.623 * |
| **Pusa Purple Round x S-324-465-2-2** | 0.53 | 0.09 | -1.62 * | -1.70 * | 0.04 | 0.04 | 0.91 | -1.86 * | 0.15 | 0.21 | -0.39 ** | -20.25 | -3.19 | -8.44 | 0.003 | 0.052 | -0.007 | 0.266 |
| **Pusa Purple Round x BLW-2001-1-1-2** | 4.44 ** | 0.57 ** | 0.58 | 0.62 | 0.25 ** | 0.30 ** | 3.70 * | 0.67 | 0.81 ** | 1.18 ** | -0.03 | 161.58 ** | 7.85 ** | 67.33 ** | 0.016 * | 0.311 * | 0.004 | 1.461 * |
| **Pant Rituraj x S-324-465-2-2** | 8.65 ** | 1.09 ** | 3.94 ** | 4.14 ** | 0.49 ** | 0.58 ** | 9.71 ** | 4.56 ** | 1.60 ** | 1.53 ** | 0.77 ** | 328.88 ** | 18.11 ** | 137.03 ** | 0.030 ** | 0.565 ** | 0.020 ** | 2.957 ** |
| **Pant Rituraj x BLW-2001-1-1-2** | -0.68 | -0.06 | -4.07 ** | -4.29 ** | -0.02 | -0.02 | -1.88 | -4.65 ** | 0.12 | 0.33 | -1.01 ** | -91.94 ** | -8.76 ** | -38.31 ** | -0.01 | -0.18 | -0.021 ** | -0.852 |
| **S-324-465-2-2 x BLW-2001-1-1-2** | 4.66 ** | 0.60 ** | -0.68 | -0.81 | 0.27 ** | 0.32 ** | 3.95 * | -0.89 | 0.84 ** | 0.98 ** | -0.22 | 110.40 ** | 4.02 * | 46.00 ** | 0.017 * | 0.320 * | -0.004 | 1.591 * |
| **SCA Compariosns** | | | | | | | | | | | | | | |  |  |  |  |
| **Sij-Sik 95%** | 4.46 | 0.56 | 2.07 | 2.19 | 0.25 | 0.30 | 4.87 | 2.35 | 0.84 | 0.86 | 0.44 | 80.51 | 5.93 | 33.55 | 0.02 | 0.372 | 0.014 | 1.829 |
| **Sij-Sik 99%** | 5.88 | 0.73 | 2.74 | 2.88 | 0.33 | 0.40 | 6.42 | 3.10 | 1.12 | 1.13 | 0.58 | 106.16 | 7.82 | 44.23 | 0.026 | 0.49 | 0.019 | 2.411 |
| **Sij-Skm 95%** | 4.21 | 0.53 | 1.96 | 2.06 | 0.23 | 0.29 | 4.59 | 2.22 | 0.79 | 0.81 | 0.41 | 75.91 | 5.59 | 31.63 | 0.019 | 0.35 | 0.013 | 1.724 |
| **Sij-Skm 99%** | 5.55 | 0.69 | 2.58 | 2.72 | 0.31 | 0.38 | 6.06 | 2.92 | 1.05 | 1.07 | 0.54 | 100.09 | 7.38 | 41.70 | 0.024 | 0.462 | 0.017 | 2.273 |

**Supplementary Table 3.** Estimation of Heterosis and heterobeltiosis for various parameters in brinjal. The symbols * and ** represent significance at P < 0.05 and P < 0.01 levels, respectively.

| **SN** | **Hybrid** | **PH** | | **NB** | | **DFF** | | **D50%F** | | **NFLC** | | **NFC** | | **FS** | | **DFH** | |
| --- | --- | --- | --- | --- | --- | --- | --- | --- | --- | --- | --- | --- | --- | --- | --- | --- | --- |
|  |  | **He** | **Hb** | **He** | **Hb** | **He** | **Hb** | **He** | **Hb** | **He** | **Hb** | **He** | **Hb** | **He** | **Hb** | **Hb** | **He** |
| **1** | **BL-2011-219-8-1 x SL-8-PB-1-3-1-4** | 39.17 ** | 31.48 ** | 41.74 ** | 34.39 ** | -7.50 ** | 39.17 ** | -7.39 ** | -10.66 ** | 25.91 ** | 21.78 ** | 45.39 ** | 37.31 ** | 40.34 ** | 32.38 ** | -7.20 ** | -10.41 ** |
| **2** | **BL-2011-219-8-1 x Pusa Purple Long** | 30.87 ** | 27.67 ** | 29.18 ** | 26.13 ** | -10.74 ** | 30.87 ** | -10.51 ** | -11.71 ** | 18.32 ** | 16.55 ** | 31.61 ** | 28.50 ** | 31.60 ** | 28.31 ** | -10.29 ** | -11.45 ** |
| **3** | **BL-2011-219-8-1 x BL-219** | 4.54 | 3.84 | 4.25 | 3.54 | -2.44 | 4.54 | -2.4 | -4.35 | 2.69 | 2.24 | 4.75 | 4.17 | 4.83 | 4.11 | -2.36 | -4.26 |
| **4** | **BL-2011-219-8-1 x Pusa Purple Round** | 11.77 | 11.18 | 11.12 | 10.62 | -0.83 | 11.77 | -0.82 | -3.56 | 6.86 | 6.55 | 11.72 | 10.98 | 12.04 | 11.44 | -0.8 | -3.47 |
| **5** | **BL-2011-219-8-1 x Pant Rituraj** | 18.07 * | 6.89 | 16.99 * | 6.44 | 6.42 * | 18.07 * | 6.35 * | 1.79 | 10.38 * | 4.08 | 18.59 * | 7.29 | 18.53 * | 7.05 | 6.18 * | 1.73 |
| **6** | **BL-2011-219-8-1 x S-324-465-2-2** | 8.28 | 4.96 | 7.78 | 4.6 | -0.04 | 8.28 | -0.04 | -0.41 | 4.88 | 2.91 | 8.62 | 5.36 | 8.47 | 5.07 | -0.05 | -0.4 |
| **7** | **BL-2011-219-8-1 x BLW-2001-1-1-2** | -0.32 | -2.17 | -0.35 | -2.07 | -4.75 | -0.32 | -4.69 | -7.27 ** | -0.22 | -1.33 | -0.2 | -2.21 | -0.32 | -2.22 | -4.59 | -7.11 ** |
| **8** | **SL-8-PB-1-3-1-4 x Pusa Purple Long** | 35.60 ** | 31.20 ** | 38.43 ** | 34.33 ** | -6.07 * | 35.60 ** | -5.90 * | -8.03 ** | 23.63 ** | 21.35 ** | 41.73 ** | 36.98 ** | 36.51 ** | 31.96 ** | -5.78 * | -7.87 ** |
| **9** | **SL-8-PB-1-3-1-4 x BL-219** | 12.86 | 7.3 | 12.03 | 6.91 | -2.29 | 12.86 | -2.26 | -7.52 ** | 7.38 | 4.29 | 13.06 | 7.34 | 13.19 | 7.47 | -2.05 | -7.21 ** |
| **10** | **SL-8-PB-1-3-1-4 x Pusa Purple Round** | 29.86 ** | 22.07 ** | 28.16 ** | 20.99 ** | -12.16 ** | 29.86 ** | -12.00 ** | -17.37 ** | 17.51 ** | 13.34 ** | 30.48 ** | 22.46 ** | 30.60 ** | 22.57 ** | -11.72 ** | -16.99 ** |
| **11** | **SL-8-PB-1-3-1-4 x Pant Rituraj** | 35.14 ** | 29.17 ** | 32.89 ** | 27.21 ** | 6.03 * | 35.14 ** | 5.95 * | 5.09 | 19.64 ** | 16.53 ** | 35.95 ** | 29.88 ** | 36.12 ** | 29.96 ** | 5.81 * | 4.98 |
| **12** | **SL-8-PB-1-3-1-4 x S-324-465-2-2** | 12.38 | 9.44 | 11.57 | 8.93 | 2.07 | 12.38 | 2.05 | -1.9 | 7.03 | 5.46 | 12.53 | 9.47 | 12.71 | 9.67 | 2 | -1.86 |
| **13** | **SL-8-PB-1-3-1-4 x BLW-2001-1-1-2** | 15.86 * | 7.54 | 14.90 * | 7.17 | -1.22 | 15.86 * | -1.2 | -7.18 ** | 9.32 * | 4.6 | 16.21 * | 7.67 | 16.07 * | 7.53 | -1.17 | -7.02 ** |
| **14** | **Pusa Purple Long x BL-219** | 23.32 ** | 21.10 ** | 29.91 ** | 27.70 ** | 2.92 | 23.32 ** | 2.97 | -0.4 | 18.72 ** | 17.45 ** | 31.92 ** | 29.50 ** | 23.88 ** | 21.60 ** | 2.88 | -0.4 |
| **15** | **Pusa Purple Long x Pusa Purple Round** | 5.57 | 2.47 | 5.64 | 2.69 | -2.68 | 5.57 | -2.74 | -6.66 * | 3.54 | 1.71 | 5.33 | 2.17 | 5.67 | 2.49 | -2.78 | -6.59 * |
| **16** | **Pusa Purple Long x Pant Rituraj** | 13.64 | 5.26 | 13.15 | 5.25 | 3.24 | 13.64 | 3.28 | 0.15 | 7.54 | 2.87 | 12.61 | 4.14 | 13.99 | 5.38 | 3.11 | 0.04 |
| **17** | **Pusa Purple Long x S-324-465-2-2** | -1.68 | -2.33 | -1.59 | -2.2 | 0.33 | -1.68 | 0.41 | -1.29 | -0.99 | -1.37 | -1.71 | -2.37 | -1.72 | -2.39 | 0.37 | -1.27 |
| **18** | **Pusa Purple Long x BLW-2001-1-1-2** | -10.33 | -14.11 * | -9.62 | -13.24 | -4.77 | -10.33 | -4.64 | -8.43 ** | -6.08 | -8.49 | -10.72 | -14.55 | -10.59 | -14.44 * | -4.46 | -8.15 ** |
| **19** | **BL-219 x Pusa Purple Round** | -2.78 | -3.95 | -2.64 | -3.75 | -9.36 ** | -2.78 | -9.25 ** | -9.98 ** | -1.66 | -2.37 | -2.83 | -4.02 | -2.85 | -4.04 | -8.92 ** | -9.64 ** |
| **20** | **BL-219 x Pant Rituraj** | 24.41 ** | 12.24 | 23.01 ** | 11.66 | 8.19 * | 24.41 ** | 8.09 * | 1.06 | 13.19 * | 6.83 | 24.35 * | 11.94 | 25.56 ** | 12.96 | 7.84 * | 1.00 |
| **21** | **BL-219 x S-324-465-2-2** | 7.69 | 5.01 | 7.27 | 4.74 | -2.96 | 7.69 | -2.93 | -4.55 | 4.5 | 2.96 | 7.84 | 5.1 | 7.88 | 5.13 | -2.74 | -4.32 |
| **22** | **BL-219 x BLW-2001-1-1-2** | -17.62 * | -19.74 ** | -16.61 * | -18.65 ** | 4.95 | -17.62 * | 4.89 | 4.1 | -10.47 * | -11.86 ** | -18.06 * | -20.20 ** | 6.82 | 4.01 | 4.75 | 3.98 |
| **23** | **Pusa Purple Round x Pant Rituraj** | 16.63 | 3.83 | 15.59 | 3.62 | 18.12 ** | 16.63 | 18.06 ** | 9.43 ** | 9.14 | 2.21 | 16.99 | 3.91 | 17.1 | 3.93 | 17.59 ** | 9.19 ** |
| **24** | **Pusa Purple Round x S-324-465-2-2** | 10.22 | 6.15 | 9.67 | 5.83 | -1.03 | 10.22 | -1.02 | -3.47 | 5.85 | 3.53 | 10.2 | 6.05 | 10.48 | 6.3 | -1.01 | -3.4 |
| **25** | **Pusa Purple Round x BLW-2001-1-1-2** | 8.32 | 6.84 | 7.9 | 6.5 | -1.74 | 8.32 | -1.72 | -1.78 | 5.02 | 4.15 | 8.47 | 6.96 | 8.51 | 6.99 | -1.69 | -1.75 |
| **26** | **Pant Rituraj x S-324-465-2-2** | 38.13 ** | 28.04 ** | 35.76 ** | 26.41 ** | 16.35 ** | 38.13 ** | 16.13 ** | 10.50 ** | 20.95 ** | 15.93 ** | 38.97 ** | 28.61 ** | 39.22 ** | 28.78 ** | 15.86 ** | 10.38 ** |
| **27** | **Pant Rituraj x BLW-2001-1-1-2** | 16.55 | 2.18 | 15.53 | 2.08 | 4.07 | 16.55 | 4.02 | -3.52 | 9.1 | 1.28 | 16.94 | 2.25 | 17.02 | 2.23 | 3.88 | -3.47 |
| **28** | **S-324-465-2-2 x BLW-2001-1-1-2** | 13.49 | 7.75 | 12.76 | 7.35 | 1.24 | 13.49 | 1.06 | -1.38 | 7.9 | 4.63 | 13.75 | 7.89 | 13.83 | 7.93 | 1.03 | -1.36 |
| **CD 5%** | | 7.057 | 7.057 | 8.149 | 0.881 | 1.018 | 3.281 | 3.457 | 3.992 | 0.394 | 0.455 | 0.481 | 0.555 | 7.706 | 8.898 | 3.719 | 4.295 |
| **CD 1%** | | 9.306 | 9.306 | 10.745 | 1.162 | 1.342 | 4.327 | 4.559 | 5.264 | 0.519 | 0.6 | 0.634 | 0.732 | 10.161 | 11.732 | 4.904 | 5.663 |

(To be continued…)

(PH – plant height, NB – number of branches per plant, DFF – days to first flowering, D50%F – days to 50% flowering, NFLC – number of flowers per flower cluster, NFC – number of fruits per cluster, FS – fruit set (%), and DFH – days to first harvest)

| **SN** | **Hybrid** | **NFPP** | | **FL** | | **FD** | | **FYPP** | | **FWT** | |
| --- | --- | --- | --- | --- | --- | --- | --- | --- | --- | --- | --- |
|  |  | **He** | **Hb** | **He** | **Hb** | **He** | **Hb** | **He** | **Hb** | **He** | **Hb** |
| **1** | **BL-2011-219-8-1 x SL-8-PB-1-3-1-4** | 45.17 ** | 38.35 ** | 33.01 ** | 27.14 ** | -17.41 ** | -23.95 ** | 53.09 ** | 32.61 ** | 6.19 | -4.13 |
| **2** | **BL-2011-219-8-1 x Pusa Purple Long** | 30.34 ** | 28.03 ** | 20.43 ** | 18.43 ** | -24.04 ** | -26.31 ** | 20.13 * | 12.45 | -7.81 | -11.94 |
| **3** | **BL-2011-219-8-1 x BL-219** | 4.81 | 4.72 | 0.09 | -4.42 | -5.27 | -9.31 | -1.19 | -1.36 | -5.16 | -5.34 |
| **4** | **BL-2011-219-8-1 x Pusa Purple Round** | 11.52 | 10.37 | 2.18 | -1.34 | -1.77 | -7.49 | 10.84 | 7.7 | -0.19 | -2.05 |
| **5** | **BL-2011-219-8-1 x Pant Rituraj** | 17.70 * | 7.61 | 12.29 | -1.7 | 15.12 * | 4.03 | 42.12 ** | 8.69 | 22.67 ** | 1.15 |
| **6** | **BL-2011-219-8-1 x S-324-465-2-2** | 8.23 | 5.67 | 3.34 | -3.52 | -0.07 | -0.9 | 11.09 | 2.22 | 2.49 | -3.28 |
| **7** | **BL-2011-219-8-1 x BLW-2001-1-1-2** | 0.23 | -2.04 | 0.2 | -6.39 | -10.18 | -15.31 ** | -8.56 | -9.57 | -8.66 | -9.76 |
| **8** | **SL-8-PB-1-3-1-4 x Pusa Purple Long** | 38.07 ** | 33.88 ** | 26.15 ** | 22.57 ** | -14.18 * | -18.68 ** | 46.89 ** | 35.13 ** | 6.31 | 0.2 |
| **9** | **SL-8-PB-1-3-1-4 x BL-219** | 12 | 6.83 | 6.92 | 6.8 | -5.17 | -16.09 ** | 9.92 | -4.92 | -0.49 | -10.01 |
| **10** | **SL-8-PB-1-3-1-4 x Pusa Purple Round** | 27.88 ** | 20.68 ** | 15.09 * | 13.89 | -27.19 ** | -36.52 ** | 7.23 | -9.33 | -14.92 * | -24.47 ** |
| **11** | **SL-8-PB-1-3-1-4 x Pant Rituraj** | 32.80 ** | 27.12 ** | 26.78 ** | 15.59 * | 14.96 * | 12.63 | 82.16 ** | 56.93 ** | 36.30 ** | 23.06 ** |
| **12** | **SL-8-PB-1-3-1-4 x S-324-465-2-2** | 11.54 | 8.8 | 6.34 | 3.74 | 4.8 | -4.23 | 21.18 * | 13.4 | 8.92 | 3.9 |
| **13** | **SL-8-PB-1-3-1-4 x BLW-2001-1-1-2** | 14.83 * | 7.08 | 5.31 | 2.8 | -2.72 | -15.10 ** | 15.18 | -1.17 | 1.35 | -7.49 |
| **14** | **Pusa Purple Long x BL-219** | 30.28 ** | 28.09 ** | 19.78 ** | 16.25 * | 6.28 | -1.17 | 61.12 ** | 50.58 ** | 23.76 ** | 18.42 ** |
| **15** | **Pusa Purple Long x Pusa Purple Round** | 3.67 | 0.81 | -1.03 | -2.85 | -6.04 | -13.99 * | -0.01 | -8.87 | -4.58 | -10.48 |
| **16** | **Pusa Purple Long x Pant Rituraj** | 12.82 | 4.86 | 8.56 | -3.56 | 7.97 | 0.35 | 29.95 ** | 4.43 | 14.63 * | -1.81 |
| **17** | **Pusa Purple Long x S-324-465-2-2** | -1.57 | -2.17 | 3.28 | -2.03 | 0.93 | -2.87 | 3.12 | 1.24 | 4.05 | 2.74 |
| **18** | **Pusa Purple Long x BLW-2001-1-1-2** | -9.69 | -13.27 | -0.37 | -5.43 | -10.24 | -17.74 ** | -17.02 * | -23.13 ** | -8.27 | -11.35 |
| **19** | **BL-219 x Pusa Purple Round** | -2.6 | -3.7 | -2.68 | -3.81 | -19.80 ** | -21.18 ** | -23.13 ** | -25.23 ** | -19.98 ** | -21.65 ** |
| **20** | **BL-219 x Pant Rituraj** | 24.27 ** | 12.73 | 25.12 ** | 13.13 | 20.59 * | 2.45 | 81.14 ** | 25.15 * | 41.26 ** | 11.45 |
| **21** | **BL-219 x S-324-465-2-2** | 6.61 | 4.13 | 6.82 | 4.26 | -6.54 | -9.93 | 7.04 | -2.42 | 0.04 | -5.74 |
| **22** | **BL-219 x BLW-2001-1-1-2** | -16.50 * | -18.52 ** | -13.01 | -15.04 * | 25.03 ** | 23.04 ** | -15.3 | -16.10 * | 4.66 | 3.58 |
| **23** | **Pusa Purple Round x Pant Rituraj** | 15.79 | 3.74 | 16.34 | 3.86 | 65.70 ** | 37.99 ** | 105.70 ** | 37.07 ** | 75.10 ** | 34.68 ** |
| **24** | **Pusa Purple Round x S-324-465-2-2** | 9.31 | 5.53 | 9.59 | 5.7 | -2.27 | -7.5 | 16.61 | 3.14 | 6.11 | -2.22 |
| **25** | **Pusa Purple Round x BLW-2001-1-1-2** | 7.49 | 6.1 | 15.49 | 11.46 | -3.63 | -3.75 | 15.07 | 13.02 | 9.75 | 6.33 |
| **26** | **Pant Rituraj x S-324-465-2-2** | 36.09 ** | 26.60 ** | 37.36 ** | 27.47 ** | 41.05 ** | 24.86 ** | 144.63 ** | 89.13 ** | 76.40 ** | 49.04 ** |
| **27** | **Pant Rituraj x BLW-2001-1-1-2** | 18.29 * | 4.48 | 18.93 * | 10.29 | 10.26 | -8.04 | 51.08 ** | 3.08 | 25.56 * | 0.31 |
| **28** | **S-324-465-2-2 x BLW-2001-1-1-2** | 12.25 | 6.93 | 12.62 | 12.55 | 2.36 | -2.98 | 28.10 ** | 15.56 | 13.48 | 8.09 |
| **CD 5%** | | 1.336 | 1.543 | 1.359 | 1.569 | 0.691 | 0.798 | 127.299 | 146.992 | 9.382 | 10.833 |
| **CD 1%** | | 1.762 | 2.035 | 1.792 | 2.069 | 0.911 | 1.052 | 167.858 | 193.826 | 12.371 | 14.285 |

(To be continued…)

(NFPP – number of fruits per plant, FL – fruit length, FD – fruit diameter, FYPP – fruit yield per plant, and FWT – fruit weight)

| **SN** | **Hybrid** | **FE** | | **TSS** | | **ACD** | | **ANTH** | |
| --- | --- | --- | --- | --- | --- | --- | --- | --- | --- |
|  |  | **He** | **Hb** | **He** | **Hb** | **He** | **Hb** | **He** | **Hb** |
| **1** | **BL-2011-219-8-1 x SL-8-PB-1-3-1-4** | 23.05 ** | 16.67 * | 33.01 ** | 27.14 ** | -17.41 ** | -23.95 ** | 53.09 ** | 32.61 ** |
| **2** | **BL-2011-219-8-1 x Pusa Purple Long** | 20.59 ** | 17.74 * | 20.43 ** | 18.43 ** | -24.04 ** | -26.31 ** | 20.13 * | 12.45 |
| **3** | **BL-2011-219-8-1 x BL-219** | 4.25 | 3.54 | 0.09 | -4.42 | -5.27 | -9.31 | -1.19 | -1.36 |
| **4** | **BL-2011-219-8-1 x Pusa Purple Round** | 11.12 | 10.62 | 2.18 | -1.34 | -1.77 | -7.49 | 10.84 | 7.7 |
| **5** | **BL-2011-219-8-1 x Pant Rituraj** | 10.48 | 6.44 | 12.29 | -1.7 | 15.12 * | 4.03 | 42.12 ** | 8.69 |
| **6** | **BL-2011-219-8-1 x S-324-465-2-2** | 7.78 | 4.6 | 3.34 | -3.52 | -0.07 | -0.9 | 11.09 | 2.22 |
| **7** | **BL-2011-219-8-1 x BLW-2001-1-1-2** | -0.35 | -2.07 | 0.2 | -6.39 | -10.18 | -15.31 ** | -8.56 | -9.57 |
| **8** | **SL-8-PB-1-3-1-4 x Pusa Purple Long** | 32.38 ** | 28.45 ** | 26.15 ** | 22.57 ** | -14.18 * | -18.68 ** | 46.89 ** | 35.13 ** |
| **9** | **SL-8-PB-1-3-1-4 x BL-219** | 12.03 | 6.91 | 6.92 | 6.8 | -5.17 | -16.09 ** | 9.92 | -4.92 |
| **10** | **SL-8-PB-1-3-1-4 x Pusa Purple Round** | 25.71 ** | 18.68 * | 15.09 * | 13.89 | -27.19 ** | -36.52 ** | 7.23 | -9.33 |
| **11** | **SL-8-PB-1-3-1-4 x Pant Rituraj** | 25.07 ** | 23.01 ** | 26.78 ** | 15.59 * | 14.96 * | 12.63 | 82.16 ** | 56.93 ** |
| **12** | **SL-8-PB-1-3-1-4 x S-324-465-2-2** | 11.57 | 8.93 | 6.34 | 3.74 | 4.8 | -4.23 | 21.18 * | 13.4 |
| **13** | **SL-8-PB-1-3-1-4 x BLW-2001-1-1-2** | 14.90 * | 7.17 | 5.31 | 2.8 | -2.72 | -15.10 ** | 15.18 | -1.17 |
| **14** | **Pusa Purple Long x BL-219** | 29.91 ** | 27.70 ** | 19.78 ** | 16.25 * | 6.28 | -1.17 | 61.12 ** | 50.58 ** |
| **15** | **Pusa Purple Long x Pusa Purple Round** | 5.64 | 2.69 | -1.03 | -2.85 | -6.04 | -13.99 * | -0.01 | -8.87 |
| **16** | **Pusa Purple Long x Pant Rituraj** | 6.69 | 5.25 | 8.56 | -3.56 | 7.97 | 0.35 | 29.95 ** | 4.43 |
| **17** | **Pusa Purple Long x S-324-465-2-2** | -1.59 | -2.2 | 3.28 | -2.03 | 0.93 | -2.87 | 3.12 | 1.24 |
| **18** | **Pusa Purple Long x BLW-2001-1-1-2** | -9.62 | -13.24 | -0.37 | -5.43 | -10.24 | -17.74 ** | -17.02 * | -23.13 ** |
| **19** | **BL-219 x Pusa Purple Round** | -2.64 | -3.75 | -2.68 | -3.81 | -19.80 ** | -21.18 ** | -23.13 ** | -25.23 ** |
| **20** | **BL-219 x Pant Rituraj** | 8.78 | 5.4 | 25.12 ** | 13.13 | 20.59 * | 2.45 | 81.14 ** | 25.15 * |
| **21** | **BL-219 x S-324-465-2-2** | 7.27 | 4.74 | 6.82 | 4.26 | -6.54 | -9.93 | 7.04 | -2.42 |
| **22** | **BL-219 x BLW-2001-1-1-2** | -8.1 | -10.35 | -13.01 | -15.04 * | 25.03 ** | 23.04 ** | -15.3 | -16.10 * |
| **23** | **Pusa Purple Round x Pant Rituraj** | 2.22 | -2.12 | 16.34 | 3.86 | 65.70 ** | 37.99 ** | 105.70 ** | 37.07 ** |
| **24** | **Pusa Purple Round x S-324-465-2-2** | 9.67 | 5.83 | 9.59 | 5.7 | -2.27 | -7.5 | 16.61 | 3.14 |
| **25** | **Pusa Purple Round x BLW-2001-1-1-2** | 7.9 | 6.5 | 15.49 | 11.46 | -3.63 | -3.75 | 15.07 | 13.02 |
| **26** | **Pant Rituraj x S-324-465-2-2** | 20.05 * | 19.15 ** | 37.36 ** | 27.47 ** | 41.05 ** | 24.86 ** | 144.63 ** | 89.13 ** |
| **27** | **Pant Rituraj x BLW-2001-1-1-2** | 2.16 | -3.5 | 18.93 * | 10.29 | 10.26 | -8.04 | 51.08 ** | 3.08 |
| **28** | **S-324-465-2-2 x BLW-2001-1-1-2** | 12.76 | 7.35 | 12.62 | 12.55 | 2.36 | -2.98 | 28.10 ** | 15.56 |
| **CD 5%** | | 1.336 | 0.031 | 0.036 | 1.569 | 0.691 | 0.798 | 127.299 | 146.992 |
| **CD 1%** | | 1.762 | 0.041 | 0.047 | 2.069 | 0.911 | 1.052 | 167.858 | 193.826 |

(FE – iron, TSS – total soluble solids, ACD– acidity, and ANTH – anthocyanin content)

**Supplementary Table 4.** Stability parameters for growth, yield, and quality traits. The symbols * and ** indicate significance at P < 0.05 and P < 0.01 levels, respectively, whereas ≠ and # denote significance at 5% and 1% levels, respectively, when tested against unity.

| **S No** | **Name of Genotypes** |  | **PH** |  |  | **NB** |  |  | **DFF** |  |  | **D50%F** |  |  | **NFLC** |  |  | **NFC** |  |  | **FS** |  |
| --- | --- | --- | --- | --- | --- | --- | --- | --- | --- | --- | --- | --- | --- | --- | --- | --- | --- | --- | --- | --- | --- | --- |
|  |  | **Mean** | **bi** | **S^2^di** | **Mean** | **bi** | **S^2^di** | **Mean** | **bi** | **S^2^di** | **Mean** | **bi** | **S^2^di** | **Mean** | **bi** | **S^2^di** | **Mean** | **bi** | **S^2^di** | **Mean** | **bi** | **S^2^di** |
| **1** | **BL-2011-219-8-1** | 56.27 | 0.7**# | -8.18 | 7.29 | 0.65**# | -0.13 | 66.17 | -0.93# | -0.61 | 70.56 | -0.9# | -0.99 | 5.12 | 0.92**# | -0.03 | 3.59 | 0.82**≠ | -0.04 | 59.8 | 0.73** | -8.92 |
| **2** | **SL-8-PB-1-3-1-4** | 50.05 | 0.69 | -4.09 | 6.53 | 0.93* | -0.08 | 61.44 | 0.54 | -0.42 | 65.58 | 0.51 | -0.47 | 4.78 | 1.02** | -0.02 | 3.19 | 0.71**# | -0.04 | 53.02 | 0.54 | -7.16 |
| **3** | **Pusa Purple Long** | 53.53 | 1.11** | -8.08 | 6.95 | 1.15** | -0.11 | 64.49 | -0.35≠ | -0.45 | 68.68 | -0.38≠ | -0.83 | 4.96 | 1.02** | -0.02 | 3.42 | 0.97** | -0.03 | 56.82 | 1.19** | -9.79 |
| **4** | **BL-219** | 55.52 | 1.1** | -8.16 | 7.19 | 1.13** | -0.12 | 68.94 | 0.96* | -1.42 | 73.49 | 0.98** | -1.55 | 5.07 | 1.02** | -0.03 | 3.55 | 1** | -0.04 | 58.98 | 1.18** | -9.82 |
| **5** | **Pusa Purple Round** | 56.87 | 1.1** | -8.21 | 7.36 | 1.12** | -0.12 | 70.09 | 0.98** | -1.43 | 74.69 | 1** | -1.56 | 5.15 | 1.02** | -0.03 | 3.64 | 1.03** | -0.04 | 60.45 | 1.18** | -9.84 |
| **6** | **Pant Rituraj** | 45.62 | 1.12** | -7.64 | 5.98 | 1.23* | -0.05 | 60.42 | 0.68** | -1.66 | 64.51 | 0.68** | -1.87 | 4.53 | 1.02** | -0.01 | 2.91 | 0.82** | -0.02 | 48.23 | 1.22** | -9.61 |
| **7** | **S-324-465-2-2** | 52.82 | 1.11** | -8.05 | 6.86 | 1.16** | -0.1 | 66.66 | 0.92* | -1.38 | 71.09 | 0.93* | -1.53 | 4.93 | 1.02** | -0.02 | 3.38 | 0.95** | -0.03 | 56.05 | 1.19** | -9.77 |
| **8** | **BLW-2001-1-1-2** | 58.45 | 1.1** | -8.25 | 7.55 | 1.1** | -0.13 | 70 | 1.01** | -1.44 | 74.6 | 1.03** | -1.57 | 5.23 | 1.02** | -0.03 | 3.74 | 1.06** | -0.04 | 62.17 | 1.17** | -9.87 |
| **9** | **BL-2011-219-8-1 x SL-8-PB-1-3-1-4** | 73.99 | 0.95** | -6.7 | 9.8 | 0.88 | -0.01 | 59.02 | 0.59 | 1.56 | 63.04 | 0.62 | 1.66 | 6.23 | 1.02** | -0.01 | 4.93 | 1.36** | 0 | 79.17 | 0.85** | -9.07 |
| **10** | **BL-2011-219-8-1 x Pusa Purple Long** | 71.85 | 1.11** | -8.34 | 9.2 | 0.99** | -0.11 | 58.31 | 1.39 | 2.47 | 62.3 | 1.4 | 2.39 | 5.96 | 1.02** | -0.03 | 4.62 | 1.32** | -0.03 | 76.74 | 1.16** | -9.96 |
| **11** | **BL-2011-219-8-1 x BL-219** | 58.43 | 1.1** | -8.25 | 7.55 | 1.1** | -0.13 | 65.91 | 1.02** | -1.41 | 70.29 | 1.03** | -1.57 | 5.23 | 1.02** | -0.03 | 3.74 | 1.06** | -0.04 | 62.26 | 1.12** | -9.91 |
| **12** | **BL-2011-219-8-1 x Pusa Purple Round** | 63.23 | 1.09** | -8.34 | 8.14 | 1.05** | -0.13 | 67.56 | 0.6 | -0.3 | 72.03 | 0.64 | -0.24 | 5.48 | 0.99** | -0.03 | 4.04 | 1.1** | -0.04 | 67.37 | 1.16** | -9.92 |
| **13** | **BL-2011-219-8-1 x Pant Rituraj** | 60.15 | 1.1** | -8.29 | 7.76 | 1.09** | -0.13 | 67.36 | 1.04** | -1.43 | 71.82 | 1.06** | -1.59 | 5.33 | 1.02** | -0.03 | 3.86 | 1.09** | -0.04 | 64.02 | 1.17** | -9.89 |
| **14** | **BL-2011-219-8-1 x S-324-465-2-2** | 59.06 | 1.16** | -8.34 | 7.63 | 1.18** | -0.13 | 66.39 | 1.09* | -1.23 | 70.8 | 1.1* | -1.39 | 5.27 | 1.04** | -0.03 | 3.79 | 1.1** | -0.04 | 62.83 | 1.23** | -9.96 |
| **15** | **BL-2011-219-8-1 x BLW-2001-1-1-2** | 57.18 | 1.1** | -8.22 | 7.4 | 1.12** | -0.12 | 64.85 | 1** | -1.39 | 69.18 | 1.01** | -1.56 | 5.16 | 1.02** | -0.03 | 3.66 | 1.03** | -0.04 | 60.79 | 1.18** | -9.85 |
| **16** | **SL-8-PB-1-3-1-4 x Pusa Purple Long** | 70.22 | 1.08** | -8.34 | 9.33 | 0.98** | -0.11 | 59.14 | 1.18* | -1.06 | 63.17 | 1.18** | -1.29 | 6.02 | 1.02** | -0.03 | 4.69 | 1.35** | -0.03 | 74.97 | 1.13** | -9.96 |
| **17** | **SL-8-PB-1-3-1-4 x BL-219** | 59.57 | 1.13** | -8.2 | 7.69 | 1.12** | -0.13 | 63.7 | 1.01* | -1.14 | 67.97 | 1.02* | -1.3 | 5.29 | 1.03** | -0.03 | 3.81 | 1.1** | -0.04 | 63.39 | 1.22** | -9.84 |
| **18** | **SL-8-PB-1-3-1-4 x Pusa Purple Round** | 69.43 | 1.02** | -7.93 | 8.9 | 0.97* | -0.09 | 57.76 | 1.18 | 0.56 | 61.72 | 1.18 | 0.4 | 5.83 | 1.02** | -0.02 | 4.46 | 1.23** | -0.03 | 74.1 | 1.03** | -9.8 |
| **19** | **SL-8-PB-1-3-1-4 x Pant Rituraj** | 64.65 | 1.09** | -8.35 | 8.31 | 1.04** | -0.13 | 64.6 | 1.13** | -1.43 | 68.92 | 1.14** | -1.62 | 5.57 | 1.02** | -0.03 | 4.15 | 1.17** | -0.04 | 68.91 | 1.15** | -9.93 |
| **20** | **SL-8-PB-1-3-1-4 x S-324-465-2-2** | 57.8 | 1.1** | -8.23 | 7.47 | 1.11** | -0.12 | 65.37 | 1.01** | -1.4 | 69.73 | 1.02** | -1.57 | 5.19 | 1.01** | -0.03 | 3.7 | 1.03** | -0.04 | 61.47 | 1.18** | -9.86 |
| **21** | **SL-8-PB-1-3-1-4 x BLW-2001-1-1-2** | 62.86 | 1.09** | -8.34 | 8.09 | 1.06** | -0.13 | 64.92 | 1.11** | -1.38 | 69.25 | 1.12** | -1.56 | 5.45 | 0.97**# | -0.03 | 4.03 | 1.14** | -0.04 | 66.85 | 1.21** | -9.88 |
| **22** | **Pusa Purple Long x BL-219** | 67.24 | 1.2** | -8.24 | 9.18 | 0.56**# | -0.13 | 68.67 | 0.96** | -1.81 | 73.2 | 0.96** | -2.03 | 5.96 | 0.89**# | -0.03 | 4.6 | 1.19** | -0.04 | 71.72 | 1.29** | -9.86 |
| **23** | **Pusa Purple Long x Pusa Purple Round** | 58.28 | 0.2 | 1.56 | 7.55 | 0.35 | 0.2 | 65.49 | 1.88 | 30.78** | 69.72 | 1.68 | 31.83** | 5.23 | 0.93** | 0.05 | 3.72 | 0.62* | -0.01 | 61.96 | -0.07≠ | -6.49 |
| **24** | **Pusa Purple Long x Pant Rituraj** | 56.34 | 0.16# | -8.02 | 7.31 | 0.1 | -0.06 | 64.48 | 1.22 | 16.31** | 68.78 | 1.11 | 18.35** | 5.11 | 0.86** | 0.01 | 3.57 | 0.55**# | -0.04 | 59.87 | 0.05# | -9.22 |
| **25** | **Pusa Purple Long x S-324-465-2-2** | 52.28 | 1.11** | -8.02 | 6.79 | 1.17** | -0.1 | 65.79 | 1.08** | -1.86 | 70.17 | 1.07** | -2.06 | 4.9 | 1.02** | -0.02 | 3.34 | 0.94** | -0.03 | 55.46 | 1.19** | -9.76 |
| **26** | **Pusa Purple Long x BLW-2001-1-1-2** | 50.2 | 0.61** | -7.36 | 6.55 | 0.75** | -0.12 | 64.03 | 1.58 | 4.18 | 68.31 | 1.5 | 4.93 | 4.79 | 0.96** | -0.03 | 3.2 | 0.66**# | -0.04 | 53.2 | 0.53**# | -9.77 |
| **27** | **BL-219 x Pusa Purple Round** | 53.98 | 1.1** | -8.1 | 7 | 1.15** | -0.11 | 62.49 | 1.13** | -1.86 | 66.7 | 1.12** | -2.07 | 4.99 | 1.02** | -0.03 | 3.45 | 0.98** | -0.04 | 57.3 | 1.19** | -9.8 |
| **28** | **BL-219 x Pant Rituraj** | 56.76 | 0.82** | -8.08 | 7.35 | 0.87** | -0.12 | 65.37 | 1.87** | -1.74 | 69.73 | 1.84** | -1.87 | 5.13 | 0.99** | -0.03 | 3.78 | 0.44 | 0.01 | 60.55 | 0.7**# | -9.85 |
| **29** | **BL-219 x S-324-465-2-2** | 56.88 | 1.1** | -8.21 | 7.36 | 1.12** | -0.12 | 64.68 | 0.97** | -1.48 | 69 | 0.96** | -1.64 | 5.15 | 1.02** | -0.03 | 3.64 | 1.03** | -0.04 | 60.46 | 1.18** | -9.84 |
| **30** | **BL-219 x BLW-2001-1-1-2** | 45.73 | 0.84**# | -8.35 | 6 | 0.99** | -0.12 | 72.35 | 1.25 | 0.12 | 77.08 | 1.23 | 0.07 | 4.54 | 0.98** | -0.03 | 2.91 | 0.69**≠ | -0.03 | 63.01 | -0.51 | 8.43 |
| **31** | **Pusa Purple Round x Pant Rituraj** | 53.21 | 1.11** | -8.07 | 6.91 | 1.16** | -0.11 | 71.37 | 1.01** | -1.75 | 76.17 | 1.1** | -2.07 | 4.95 | 1.02** | -0.02 | 3.4 | 0.96** | -0.03 | 56.47 | 1.19** | -9.78 |
| **32** | **Pusa Purple Round x S-324-465-2-2** | 58.22 | 1.1** | -8.25 | 7.52 | 1.11** | -0.13 | 65.97 | 1.14** | -1.85 | 70.36 | 1.14** | -2.06 | 5.21 | 1.03** | -0.03 | 3.72 | 1.06** | -0.04 | 61.92 | 1.17** | -9.86 |
| **33** | **Pusa Purple Round x BLW-2001-1-1-2** | 61.61 | 1.09** | -8.32 | 7.94 | 1.07** | -0.13 | 68.78 | 1.17** | -1.82 | 73.32 | 1.17** | -2.03 | 5.4 | 1.02** | -0.03 | 3.95 | 1.12** | -0.04 | 65.6 | 1.16** | -9.9 |
| **34** | **Pant Rituraj x S-324-465-2-2** | 63.02 | 1.09** | -8.34 | 8.11 | 1.06** | -0.13 | 70.3 | 1.07** | -1.86 | 74.92 | 1.07** | -2.07 | 5.46 | 0.96**# | -0.03 | 4.04 | 1.14** | -0.04 | 67.14 | 1.16** | -9.92 |
| **35** | **Pant Rituraj x BLW-2001-1-1-2** | 53.17 | 1.11** | -8.33 | 6.9 | 1.19** | -0.12 | 62.88 | 1.96** | -1.8 | 67.11 | 1.92** | -1.94 | 4.94 | 1.03** | -0.03 | 3.4 | 0.96** | -0.04 | 56.43 | 1.17** | -9.96 |
| **36** | **S-324-465-2-2 x BLW-2001-1-1-2** | 59.95 | 1.1** | -8.29 | 7.73 | 1.09** | -0.13 | 67.49 | 1** | -1.8 | 71.84 | 1.14** | -2.02 | 5.31 | 1.02** | -0.03 | 3.84 | 1.09** | -0.04 | 63.8 | 1.17** | -9.89 |
| **37** | **Check (Kalpataru)** | 61.55 | 1.15** | -8.21 | 7.93 | 1.12** | -0.13 | 69.66 | 0.57**# | -1.87 | 74.24 | 0.59**# | -2.08 | 5.4 | 1.02** | -0.03 | 3.95 | 1.14** | -0.04 | 65.54 | 1.24** | -9.85 |
| **Population Mean** | | 58.54 |  |  |  | 7.6 |  |  | 65.48 |  |  | 69.84 |  |  | 5.25 |  |  | 3.77 |  |  | 62.67 |  |
| **SE** | | 2.04 | 0.15 |  | 0.25 | 0.35 |  | 0.97 | 0.79 |  | 1.02 | 0.75 |  | 0.12 | 0.12 |  | 0.14 | 0.11 |  | 2.23 | 0.19 |  |
| **CD (5%)** | | 5.68 |  |  | 0.71 |  |  | 2.68 |  |  | 2.83 |  |  | 0.34 |  |  | 0.39 |  |  | 6.2 |  |  |

(To be continued….)

(PH – plant height, NB – number of branches per plant, DFF – days to first flowering, D50%F – days to 50% flowering, NFLC – number of flowers per flower cluster, NFC – number of fruits per cluster and FS – fruit set (%))

| **S No** | **Name of Genotypes** |  | **DFFH** |  |  | | **NFPP** |  |  | **FL** |  |  | **FD** |  |  | **FYPP** |  |  | **FWT** |  |  | **FY. ha** |  |
| --- | --- | --- | --- | --- | --- | --- | --- | --- | --- | --- | --- | --- | --- | --- | --- | --- | --- | --- | --- | --- | --- | --- | --- |
|  |  | **Mean** | **bi** | **S^2^di** | **Mean** | **bi** | | **S^2^di** | **Mean** | **bi** | **S^2^di** | **Mean** | **bi** | **S^2^di** | **Mean** | **bi** | **S^2^di** | **Mean** | **bi** | **S^2^di** | **Mean** | **bi** | **S^2^di** |
| **1** | **BL-2011-219-8-1** | 77.84 | -0.65 | 5.61 | 10.55 | | 0.7**# | -0.3 | 11.4 | 1.07* | -0.13 | 6.28 | 0.62 | 0.47** | 870.48 | 0.86* | 8686.24* | 82.54 | 0.89 | 103.3** | 362.7 | 0.87* | 1509.09* |
| **2** | **SL-8-PB-1-3-1-4** | 72.45 | 0.14**# | -2.4 | 9.62 | | 0.98** | -0.09 | 10.39 | 1.57** | -0.31 | 5.28 | 1.09** | 0.02 | 634.95 | 0.93**# | -2721.85 | 66.5 | 1.11** | -8.49 | 264.67 | 0.94**# | -472.05 |
| **3** | **Pusa Purple Long** | 75.82 | 0.59 | 7.53* | 10.24 | | 1.04** | -0.28 | 11.02 | 0.85**# | -0.31 | 5.9 | 0.37 | 0.01 | 759.2 | 0.7** | -725.66 | 75.14 | 0.54*≠ | -5.51 | 316.33 | 0.7** | -125.92 |
| **4** | **BL-219** | 80.98 | 1.31** | -2.02 | 10.6 | | 1.04** | -0.28 | 10.37 | 0.86**≠ | -0.31 | 6.86 | 0.86** | -0.07 | 873.45 | 1.05** | -2548.33 | 82.24 | 0.87** | -13.25 | 363.94 | 1.05** | -442.43 |
| **5** | **Pusa Purple Round** | 82.28 | 1.32** | -2.02 | 10.84 | | 1.05** | -0.29 | 10.61 | 0.87**≠ | -0.31 | 7.11 | 0.87** | -0.07 | 919.67 | 0.96**# | -2722.21 | 85.74 | 0.9** | -13.28 | 384.44 | 0.99**# | -472.72 |
| **6** | **Pant Rituraj** | 71.31 | 0.98** | -1.96 | 8.8 | | 0.97** | -0.27 | 8.56 | 0.82** | -0.3 | 5.07 | 0.82**# | -0.08 | 460.99 | 0.53**# | -2493.16 | 53.58 | 0.66**# | -14.33 | 192.08 | 0.53**# | -432.99 |
| **7** | **S-324-465-2-2** | 78.39 | 1.28** | -2.01 | 10.12 | | 1.03** | -0.28 | 9.88 | 0.85**# | -0.31 | 6.38 | 0.84** | -0.07 | 731.55 | 0.81**# | -2705.82 | 73.26 | 0.8**# | -14.24 | 304.81 | 0.82**# | -469.94 |
| **8** | **BLW-2001-1-1-2** | 82.18 | 1.34** | -2.09 | 11.12 | | 1.05** | -0.29 | 9.9 | 0.88** | -0.31 | 7.09 | 0.88** | -0.07 | 890.26 | 0.95**# | -2720.07 | 80.56 | 0.88** | -13.55 | 370.94 | 0.95**# | -472.52 |
| **9** | **BL-2011-219-8-1 x SL-8-PB-1-3-1-4** | 69.73 | 1.56 | 0.24 | 14.69 | | 0.91** | -0.19 | 14.49 | 1.45** | -0.28 | 4.77 | 0.52**# | -0.07 | 1154.37 | 1** | -1011.33 | 79.14 | 0.83**# | -14.25 | 480.99 | 1.01** | -175.65 |
| **10** | **BL-2011-219-8-1 x Pusa Purple Long** | 68.92 | 2.14** | -2.32 | 13.6 | | 0.99** | -0.26 | 13.5 | 1.21** | -0.26 | 4.63 | 0.72 | 0.07 | 978.86 | 0.96** | 6560.4 | 72.68 | 0.88* | 23.95 | 407.86 | 0.97** | 1139.27 |
| **11** | **BL-2011-219-8-1 x BL-219** | 77.53 | 1.33** | -2.24 | 11.12 | | 1.05** | -0.29 | 10.89 | 0.88** | -0.31 | 6.23 | 0.88** | -0.07 | 861.58 | 0.95**# | -2720.34 | 78.14 | 0.88** | -13.09 | 358.99 | 0.95**# | -471.55 |
| **12** | **BL-2011-219-8-1 x Pusa Purple Round** | 79.42 | 1.35 | 0.06 | 11.97 | | 1.05** | -0.29 | 11.24 | 0.64**# | -0.31 | 6.57 | 0.64**# | -0.08 | 993.75 | 0.89**# | -2707.63 | 83.99 | 0.67**# | -14.6 | 414.06 | 0.89**# | -470.07 |
| **13** | **BL-2011-219-8-1 x Pant Rituraj** | 79.18 | 1.34** | -2.24 | 11.43 | | 1.06** | -0.29 | 11.2 | 0.89** | -0.31 | 6.53 | 0.89** | -0.07 | 946.13 | 1.03** | -2687.53 | 83.49 | 0.92** | -12.5 | 394.22 | 1.04** | -467.56 |
| **14** | **BL-2011-219-8-1 x S-324-465-2-2** | 78.08 | 1.42** | -2.27 | 11.22 | | 1.07** | -0.29 | 10.99 | 0.88** | -0.3 | 6.33 | 0.87** | -0.06 | 889.84 | 0.98** | -2550.1 | 79.84 | 0.88** | -9.41 | 370.77 | 0.98** | -442.94 |
| **15** | **BL-2011-219-8-1 x BLW-2001-1-1-2** | 76.33 | 1.31** | -2.24 | 10.9 | | 1.05** | -0.29 | 10.67 | 0.87**≠ | -0.31 | 6 | 0.87** | -0.07 | 805.03 | 0.9**# | -2720.54 | 74.49 | 0.85** | -13.78 | 332.1 | 0.84**# | -471.66 |
| **16** | **SL-8-PB-1-3-1-4 x Pusa Purple Long** | 69.85 | 1.48** | -2.4 | 13.71 | | 1.1** | -0.29 | 13.5 | 0.9** | -0.29 | 4.8 | 0.89** | -0.05 | 1025.92 | 1.12** | -1831.86 | 75.29 | 0.97** | -8.28 | 427.46 | 1.13** | -317.93 |
| **17** | **SL-8-PB-1-3-1-4 x BL-219** | 75.14 | 1.31** | -2.35 | 11.32 | | 1.06** | -0.28 | 11.1 | 0.85** | -0.3 | 5.76 | 0.84** | -0.07 | 830.48 | 0.9**# | -2682.28 | 74.01 | 0.83** | -12.26 | 346.04 | 0.9**# | -465.06 |
| **18** | **SL-8-PB-1-3-1-4 x Pusa Purple Round** | 68.3 | 1.77** | -2.29 | 13.08 | | 1.08** | -0.3 | 12.09 | 0.78** | -0.26 | 4.51 | 0.76* | -0.02 | 836.56 | 0.83** | 3.93 | 64.76 | 0.73** | -0.47 | 348.57 | 0.83** | 1.21 |
| **19** | **SL-8-PB-1-3-1-4 x Pant Rituraj** | 76.05 | 1.38** | -2.38 | 12.23 | | 1.07** | -0.3 | 12.01 | 0.92** | -0.3 | 5.95 | 0.91** | -0.06 | 1000.59 | 1.09** | -2433.71 | 81.84 | 0.96** | -11.03 | 416.91 | 1.09** | -422.9 |
| **20** | **SL-8-PB-1-3-1-4 x S-324-465-2-2** | 76.93 | 1.32** | -2.24 | 11.01 | | 1.05** | -0.29 | 10.78 | 0.88** | -0.31 | 6.11 | 0.87** | -0.07 | 829.56 | 0.91**# | -2719.49 | 76.11 | 0.86** | -13.4 | 345.65 | 0.92**# | -471.88 |
| **21** | **SL-8-PB-1-3-1-4 x BLW-2001-1-1-2** | 76.41 | 1.38** | -2.36 | 11.91 | | 1.06** | -0.29 | 10.68 | 0.91** | -0.3 | 6.02 | 0.9** | -0.06 | 879.87 | 0.96** | -2533 | 74.52 | 0.88** | -11.74 | 366.61 | 0.96** | -439.55 |
| **22** | **Pusa Purple Long x BL-219** | 80.66 | 0.89**# | -2.4 | 13.58 | | 0.8**# | -0.3 | 12.81 | 1.04** | -0.29 | 6.78 | 1.05** | -0.07 | 1315.24 | 1.28** | -2263.83 | 97.39 | 1.21** | -11.33 | 548.02 | 1.29** | -392.96 |
| **23** | **Pusa Purple Long x Pusa Purple Round** | 76.85 | -1.02 | 20.84** | 10.93 | | 0.67 | 0.36 | 10.7 | 2.06** | -0.01 | 6.11 | 2.3** | 0.17 | 840.86 | 1.59** | -1953.33 | 76.76 | 2.25** | 68.05* | 350.36 | 1.6** | -338.71 |
| **24** | **Pusa Purple Long x Pant Rituraj** | 75.86 | -0.84 | 6.78 | 10.74 | | 0.69** | -0.09 | 10.62 | 1.59** | -0.23 | 5.92 | 1.88** | 0.19 | 792.85 | 1.3** | -2187.1 | 73.78 | 1.77** | 35.53 | 330.35 | 1.31** | -378.22 |
| **25** | **Pusa Purple Long x S-324-465-2-2** | 77.39 | 1** | -2.36 | 10.02 | | 1.03** | -0.28 | 10.79 | 0.84**# | -0.31 | 6.2 | 1.05** | -0.08 | 768.61 | 0.96** | -2619.39 | 77.2 | 0.99** | -14.97 | 320.25 | 0.96** | -454.6 |
| **26** | **Pusa Purple Long x BLW-2001-1-1-2** | 75.47 | 0.14 | 3.14 | 9.65 | | 0.9** | -0.19 | 10.42 | 1.43** | -0.28 | 5.83 | 1.65** | -0.05 | 684.34 | 1.13** | -2721.33 | 71.41 | 1.5** | -2.97 | 286.81 | 1.11** | -459.25 |
| **27** | **BL-219 x Pusa Purple Round** | 73.76 | 0.95** | -2.01 | 10.32 | | 1.04** | -0.28 | 10.09 | 0.85**# | -0.31 | 5.51 | 1.06** | -0.08 | 671.43 | 0.83**# | -2703.22 | 65.81 | 0.9**# | -14.87 | 279.76 | 0.84**# | -469.67 |
| **28** | **BL-219 x Pant Rituraj** | 76.9 | 1.35 | 0.86 | 10.93 | | 1.12** | -0.28 | 10.7 | 1.15** | -0.26 | 6.11 | 1.34** | -0.03 | 835.04 | 1.17** | -940.45 | 75.69 | 1.27** | -2.68 | 347.93 | 1.17** | -163.28 |
| **29** | **BL-219 x S-324-465-2-2** | 76.24 | 0.83** | -2.26 | 10.79 | | 0.97** | -0.29 | 10.56 | 0.9** | -0.3 | 5.97 | 1.11** | -0.07 | 783.08 | 0.94** | -2213.77 | 73.29 | 1.01** | -12.39 | 326.28 | 0.95** | -383.78 |
| **30** | **BL-219 x BLW-2001-1-1-2** | 84.82 | 0.55 | -1.29 | 8.85 | | 0.94** | -0.29 | 8.61 | 1.13** | -0.3 | 8.58 | 1.34** | -0.07 | 739.85 | 1.02** | -2606.95 | 84.31 | 1.26** | -11.72 | 308.27 | 1.03** | -452.72 |
| **31** | **Pusa Purple Round x Pant Rituraj** | 83.85 | 1.04** | -2.4 | 10.19 | | 1.04** | -0.28 | 9.95 | 0.85**# | -0.31 | 8.4 | 1.05** | -0.08 | 948.27 | 1.09** | -2540.27 | 93.82 | 1.03** | -14.78 | 395.11 | 1.09** | -441.38 |
| **32** | **Pusa Purple Round x S-324-465-2-2** | 77.6 | 1.15** | -2.38 | 11.06 | | 1.06** | -0.28 | 10.83 | 0.82** | -0.3 | 6.24 | 1.02** | -0.08 | 853.06 | 0.98**# | -2721.52 | 77.73 | 0.94** | -13.62 | 353.78 | 0.95**# | -471.09 |
| **33** | **Pusa Purple Round x BLW-2001-1-1-2** | 80.79 | 1.22** | -2.4 | 11.65 | | 1.08** | -0.27 | 11.43 | 0.81** | -0.3 | 6.83 | 1.01** | -0.07 | 1023.11 | 1.12** | -2720.68 | 88.41 | 0.99** | -12.46 | 426.85 | 1.14** | -472.59 |
| **34** | **Pant Rituraj x S-324-465-2-2** | 82.62 | 0.96** | -2.34 | 11.97 | | 1.04** | -0.28 | 11.75 | 0.84**# | -0.31 | 7.15 | 1.04** | -0.08 | 1127.72 | 1.23** | -2588.96 | 94.52 | 1.07** | -14.91 | 469.88 | 1.23** | -448.4 |
| **35** | **Pant Rituraj x BLW-2001-1-1-2** | 74.08 | 1.43 | 1.61 | 10.41 | | 1.14** | -0.28 | 10.17 | 1.16** | -0.25 | 5.59 | 1.35** | -0.02 | 696.46 | 1.03** | -376.65 | 67.28 | 1.17** | -0.21 | 286.86 | 0.97** | 2.24 |
| **36** | **S-324-465-2-2 x BLW-2001-1-1-2** | 79.19 | 1.2** | -2.4 | 11.36 | | 1.08** | -0.27 | 11.13 | 0 .8** | -0.3 | 6.53 | 1** | -0.07 | 937.1 | 1.05** | -2720.79 | 83.13 | 0.96** | -13.15 | 390.46 | 1.06** | -472.09 |
| **37** | **Check (Kalpataru)** | 81.79 | 0.76 | -1.55 | 11.55 | | 0.93** | -0.28 | 10.33 | 0.86** | -0.28 | 7.02 | 0.87** | -0.06 | 945.47 | 0.94** | -1125.78 | 82.61 | 0.88** | -8.59 | 393.94 | 0.95** | -194.89 |
| **Population Mean** | | 77.05 |  |  | 11.19 | |  |  | 10.98 |  |  | 6.19 |  |  | 868.53 |  |  | 77.86 |  |  | 361.76 |  |  |
| **SE** | | 1.1 | 0.69 |  | 0.39 | | 0.12 |  | 0.39 | 0.21 |  | 0.2 | 0.27 |  | 36.9 | 0.12 |  | 2.74 | 0.23 |  | 15.37 | 0.12 |  |
| **CD (5%)** | | 3.04 |  |  | 1.08 | |  |  | 1.09 |  |  | 0.56 |  |  | 102.44 |  |  | 7.61 |  |  | 42.68 |  |  |

(To be continued….)

(DFH – days to first harvest, NFPP – number of fruits per plant, FL – fruit length, FD – fruit diameter, FYPP – fruit yield per plant, FWT – fruit weight and, FY. ha- fruit yield per hectare)

| **S No** | **Name of Genotypes** |  | **FE** |  |  | **TSS** |  |  | **ACD** |  |  | **ANTH** |  |
| --- | --- | --- | --- | --- | --- | --- | --- | --- | --- | --- | --- | --- | --- |
|  |  | **Mean** | **bi** | **S^2^di** | **Mean** | **bi** | **S^2^di** | **Mean** | **bi** | **S^2^di** | **Mean** | **bi** | **S^2^di** |
| **1** | **BL-2011-219-8-1** | 0.24 | 0.7**# | 0 | 5.78 | 0.68**≠ | -0.06 | 0.3 | 0.17# | 0 | 16.77 | 0.47**# | -1.4 |
| **2** | **SL-8-PB-1-3-1-4** | 0.21 | 0.96 | 0 | 5.32 | 1.05 | -0.04 | 0.28 | 0.65**# | 0 | 14.66 | 0.78** | -1.33 |
| **3** | **Pusa Purple Long** | 0.23 | 1.26** | 0 | 5.57 | 1.25** | -0.05 | 0.29 | 0.61 | 0 | 15.72 | 1.19** | -1.39 |
| **4** | **BL-219** | 0.23 | 1.24** | 0 | 5.72 | 1.23** | -0.06 | 0.32 | 1.13** | 0 | 16.81 | 1.53** | -1.3 |
| **5** | **Pusa Purple Round** | 0.24 | 1.22** | 0 | 5.82 | 1.22** | -0.06 | 0.32 | 1.15** | 0 | 16.99 | 1.15** | -1.4 |
| **6** | **Pant Rituraj** | 0.22 | 0.28# | 0 | 5.45 | 0.23≠ | -0.05 | 0.27 | 0.9** | 0 | 15.13 | 0.44**# | -1.4 |
| **7** | **S-324-465-2-2** | 0.22 | 1.27** | 0 | 5.52 | 1.25** | -0.05 | 0.3 | 1.09** | 0 | 15.45 | 1.2** | -1.38 |
| **8** | **BLW-2001-1-1-2** | 0.25 | 1.2** | 0 | 5.94 | 1.2** | -0.06 | 0.32 | 1.16** | 0 | 17.59 | 1.13** | -1.4 |
| **9** | **BL-2011-219-8-1 x SL-8-PB-1-3-1-4** | 0.28 | 0.14 | 0 | 6.55 | -0.09# | -0.05 | 0.26 | 1.04* | 0 | 20.59 | 0.51 | -0.32 |
| **10** | **BL-2011-219-8-1 x Pusa Purple Long** | 0.28 | 1.41** | 0 | 6.58 | 1.39** | -0.05 | 0.26 | 1.35** | 0 | 20.78 | 1.39** | -1.3 |
| **11** | **BL-2011-219-8-1 x BL-219** | 0.25 | 1.2** | 0 | 5.94 | 1.2** | -0.06 | 0.3 | 1.12** | 0 | 17.58 | 1.13** | -1.4 |
| **12** | **BL-2011-219-8-1 x Pusa Purple Round** | 0.27 | 1.13** | 0 | 6.31 | 1.16** | -0.06 | 0.31 | 1.06** | 0 | 19.39 | 1.07** | -1.35 |
| **13** | **BL-2011-219-8-1 x Pant Rituraj** | 0.25 | 1.17** | 0 | 6.04 | 1.35** | -0.05 | 0.31 | 1.14** | 0 | 18.05 | 1.29** | -1.38 |
| **14** | **BL-2011-219-8-1 x S-324-465-2-2** | 0.25 | 1.28** | 0 | 5.98 | 1.2** | -0.06 | 0.3 | 1.16** | 0 | 17.76 | 1.12** | -1.39 |
| **15** | **BL-2011-219-8-1 x BLW-2001-1-1-2** | 0.24 | 1.21** | 0 | 5.85 | 1.22** | -0.06 | 0.29 | 1.1** | 0 | 17.1 | 1.14** | -1.4 |
| **16** | **SL-8-PB-1-3-1-4 x Pusa Purple Long** | 0.29 | 2.36 | 0 | 6.79 | 2.25 | 0.13 | 0.26 | 1.13** | 0 | 21.73 | 2.37** | 0.55 |
| **17** | **SL-8-PB-1-3-1-4 x BL-219** | 0.25 | 1.22** | 0 | 6.03 | 1.23** | -0.06 | 0.29 | 1.11** | 0 | 18.01 | 1.15** | -1.4 |
| **18** | **SL-8-PB-1-3-1-4 x Pusa Purple Round** | 0.29 | 0.1≠ | 0 | 6.67 | 0.08 | -0.04 | 0.24 | 0.5**# | 0 | 21.21 | 0.35**# | -1.4 |
| **19** | **SL-8-PB-1-3-1-4 x Pant Rituraj** | 0.27 | 1.11** | 0 | 6.41 | 1.08** | -0.06 | 0.29 | 1.14** | 0 | 19.78 | 0.89** | -1.39 |
| **20** | **SL-8-PB-1-3-1-4 x S-324-465-2-2** | 0.24 | 1.2** | 0 | 5.89 | 1.21** | -0.06 | 0.3 | 1.11** | 0 | 17.34 | 1.14** | -1.4 |
| **21** | **SL-8-PB-1-3-1-4 x BLW-2001-1-1-2** | 0.26 | 1.14** | 0 | 6.25 | 1.28** | -0.06 | 0.29 | 1.14** | 0 | 19.13 | 1.2** | -1.4 |
| **22** | **Pusa Purple Long x BL-219** | 0.3 | 0.6**# | 0 | 6.95 | 0.57**# | -0.06 | 0.31 | 1.01** | 0 | 22.61 | 0.78**≠ | -1.36 |
| **23** | **Pusa Purple Long x Pusa Purple Round** | 0.25 | 0.28 | 0 | 5.94 | 0.4 | 0.09 | 0.3 | 0.6 | 0** | 17.66 | 0.1 | 0.96 |
| **24** | **Pusa Purple Long x Pant Rituraj** | 0.24 | 0.06 | 0 | 5.8 | 0.07 | -0.02 | 0.29 | 0.53 | 0 | 16.82 | 0.22# | -1.23 |
| **25** | **Pusa Purple Long x S-324-465-2-2** | 0.22 | 1.28** | 0 | 5.48 | 1.26** | -0.05 | 0.3 | 1.04** | 0 | 15.36 | 1.19** | -1.22 |
| **26** | **Pusa Purple Long x BLW-2001-1-1-2** | 0.21 | 0.78* | 0 | 5.33 | 0.82 | -0.05 | 0.29 | 0.89 | 0 | 14.6 | 0.85** | -1.1 |
| **27** | **BL-219 x Pusa Purple Round** | 0.23 | 1.26** | 0 | 5.6 | 1.24** | -0.06 | 0.28 | 1.02** | 0 | 15.89 | 1.18** | -1.39 |
| **28** | **BL-219 x Pant Rituraj** | 0.24 | 0.93** | 0 | 5.82 | 0.96** | -0.05 | 0.3 | 1.29** | 0 | 16.74 | 1** | -1.17 |
| **29** | **BL-219 x S-324-465-2-2** | 0.24 | 1.22** | 0 | 5.82 | 1.22** | -0.06 | 0.29 | 0.92** | 0 | 16.88 | 1.26** | -1.38 |
| **30** | **BL-219 x BLW-2001-1-1-2** | 0.22 | -0.63# | 0 | 5.36 | -0.74# | -0.04 | 0.33 | 1** | 0 | 14.66 | -0.21# | -1.32 |
| **31** | **Pusa Purple Round x Pant Rituraj** | 0.23 | 1.27** | 0 | 5.55 | 1.25** | -0.05 | 0.33 | 1.1** | 0 | 15.49 | 1.07** | -1.34 |
| **32** | **Pusa Purple Round x S-324-465-2-2** | 0.25 | 1.2** | 0 | 5.93 | 1.21** | -0.06 | 0.3 | 0.76** | 0 | 17.5 | 1.13** | -1.4 |
| **33** | **Pusa Purple Round x BLW-2001-1-1-2** | 0.26 | 1.15** | 0 | 6.18 | 1.18** | -0.06 | 0.31 | 1.14** | 0 | 18.67 | 0.97** | -1.4 |
| **34** | **Pant Rituraj x S-324-465-2-2** | 0.27 | 1.13** | 0 | 6.29 | 1.16** | -0.06 | 0.32 | 1.08** | 0 | 19.42 | 1.07** | -1.4 |
| **35** | **Pant Rituraj x BLW-2001-1-1-2** | 0.23 | 1.3** | 0 | 5.54 | 1.3** | -0.06 | 0.28 | 1.31** | 0 | 15.59 | 1.19** | -1.4 |
| **36** | **S-324-465-2-2 x BLW-2001-1-1-2** | 0.25 | 1.17** | 0 | 6.06 | 1.19** | -0.06 | 0.31 | 1.12** | 0 | 18.15 | 1.11** | -1.39 |
| **37** | **Check (Kalpataru)** | 0.26 | 1.21** | 0 | 6.18 | 1.23** | -0.06 | 0.32 | 0.91** | 0 | 18.75 | 1.15** | -1.39 |
| **Population Mean** | | 0.25 |  |  | 5.95 |  |  | 0.3 |  |  | 17.63 |  |  |
| **SE** | | 0.01 | 0.39 |  | 0.17 | 0.56 |  | 0.01 | 0.45 |  | 0.84 | 0.22 |  |
| **CD (5%)** | | 0.03 |  |  | 0.48 |  |  | 0.02 |  |  | 2.32 |  |  |

(FE – iron, TSS – total soluble solids, ACD– acidity, and ANTH – anthocyanin content)

**Supplementary Table 5.** Best stable crosses for different environments

| **Characters** | **Better environment** | **Poor environment** | **Average environment** |
| --- | --- | --- | --- |
| Plant height (cm) at 90 DAT | BL-2011-219-8-1 x Pusa Purple Long | BL-2011-219-8-1 x SL-8-PB-1-3-1-4 |  |
|  | SL-8-PB-1-3-1-4 x Pusa Purple Long |  |  |
|  | SL-8-PB-1-3-1-4 x Pusa Purple Round |  |  |
| Number of branches at 90 DAT | SL-8-PB-1-3-1-4 x Pant Rituraj | BL-2011-219-8-1 x Pusa Purple Long |  |
|  | BL-2011-219-8-1 x Pusa Purple Round | SL-8-PB-1-3-1-4 x Pusa Purple Long |  |
|  | Pant Rituraj x S-324-465-2-2 | Pusa Purple Long x BL-219 |  |
| Days to first flowering | SL-8-PB-1-3-1-4 x Pusa Purple Long | BL-219 x S-324-465-2-2 | BL-2011-219-8-1 x BLW-2001-1-1-2 |
|  | BL-219 x Pusa Purple Round |  |  |
|  | Pant Rituraj x BLW-2001-1-1-2 |  |  |
| Days to 50% flowering | SL-8-PB-1-3-1-4 x Pusa Purple Long | BL-219 x S-324-465-2-2 |  |
|  | BL-219 x Pusa Purple Round |  |  |
|  | Pant Rituraj x BLW-2001-1-1-2 |  |  |
| Number of flowers per cluster | SL-8-PB-1-3-1-4 x Pusa Purple Long | BL-2011-219-8-1 x Pusa Purple Round |  |
|  | SL-8-PB-1-3-1-4 x Pusa Purple Round | Pusa Purple Long x BL-219 |  |
|  | SL-8-PB-1-3-1-4 x Pant Rituraj | Pant Rituraj x S-324-465-2-2 |  |
| Number of fruit per cluster | BL-2011-219-8-1 x SL-8-PB-1-3-1-4 |  |  |
|  | SL-8-PB-1-3-1-4 x Pusa Purple Long |  |  |
|  | BL-2011-219-8-1 x Pusa Purple Long |  |  |
| Fruit setting (%) | BL-2011-219-8-1 x Pusa Purple Long | BL-2011-219-8-1 x SL-8-PB-1-3-1-4 |  |
|  | SL-8-PB-1-3-1-4 x Pusa Purple Long |  |  |
|  | SL-8-PB-1-3-1-4 x Pusa Purple Round |  |  |
| Days to first harvest | BL-2011-219-8-1 x P  usa Purple Long | BL-219 x Pusa Purple Round |  |
|  | SL-8-PB-1-3-1-4 x Pusa Purple Long | BL-219 x S-324-465-2-2 |  |
|  | SL-8-PB-1-3-1-4 x Pusa Purple Round |  |  |
| Number of fruits per plant | SL-8-PB-1-3-1-4 x Pusa Purple Long | Pusa Purple Long x BL-219 |  |
|  | SL-8-PB-1-3-1-4 x Pusa Purple Round | BL-2011-219-8-1 x SL-8-PB-1-3-1-4 |  |
|  | SL-8-PB-1-3-1-4 x Pant Rituraj | BL-2011-219-8-1 x Pusa Purple Long |  |
| Fruit length (cm) | BL-2011-219-8-1 x SL-8-PB-1-3-1-4 | SL-8-PB-1-3-1-4 x Pusa Purple Long |  |
|  | Pusa Purple Long x BL-219 | SL-8-PB-1-3-1-4 x Pusa Purple Round |  |
|  | BL-2011-219-8-1 x Pusa Purple Long | SL-8-PB-1-3-1-4 x Pant Rituraj |  |
| Fruit diameter (cm^2^) | BL-219 x BLW-2001-1-1-2 | BL-2011-219-8-1 x Pusa Purple Round | S-324-465-2-2 x BLW-2001-1-1-2 |
|  | Pusa Purple Round x Pant Rituraj | BL-2011-219-8-1 x Pant Rituraj |  |
|  | Pant Rituraj x S-324-465-2-2 | BL-2011-219-8-1 x S-324-465-2-2 |  |
| Fruit yield plant^-1^ | Pusa Purple Long x BL-219 | BL-2011-219-8-1 x Pusa Purple Long | BL-2011-219-8-1 x SL-8-PB-1-3-1-4 |
|  | SL-8-PB-1-3-1-4 x Pant Rituraj | BL-2011-219-8-1 x Pusa Purple Round |  |
|  | SL-8-PB-1-3-1-4 x Pusa Purple Long | BL-2011-219-8-1 x Pant Rituraj |  |
| Average fruit weight (g) | Pusa Purple Long x BL-219 | BL-2011-219-8-1 x Pusa Purple Round |  |
|  | Pant Rituraj x S-324-465-2-2 | BL-2011-219-8-1 x Pant Rituraj |  |
|  | Pusa Purple Round x Pant Rituraj | Pusa Purple Round x BLW-2001-1-1-2 |  |
| Fruit yield (qha^-1^) | Pusa Purple Long x BL-219 | BL-2011-219-8-1 x Pusa Purple Long |  |
|  | BL-2011-219-8-1 x SL-8-PB-1-3-1-4 | BL-2011-219-8-1 x Pusa Purple Round |  |
|  | Pant Rituraj x S-324-465-2-2 | BL-2011-219-8-1 x S-324-465-2-2 |  |
| Iron (mg100g^-1^) | BL-2011-219-8-1 x SL-8-PB-1-3-1-4 | Pusa Purple Long x BL-219 |  |
|  | BL-2011-219-8-1 x Pusa Purple Round | SL-8-PB-1-3-1-4 x Pusa Purple Round |  |
|  | SL-8-PB-1-3-1-4 x Pant Rituraj | BL-2011-219-8-1 x SL-8-PB-1-3-1-4 |  |
| Total soluble solid (^O^B) | BL-2011-219-8-1 x Pusa Purple Long | Pusa Purple Long x BL-219 |  |
|  | BL-2011-219-8-1 x Pusa Purple Round |  |  |
|  | SL-8-PB-1-3-1-4 x Pant Rituraj |  |  |
| Acidity (%) | BL-2011-219-8-1 x SL-8-PB-1-3-1-4 | SL-8-PB-1-3-1-4 x Pusa Purple Round |  |
|  | BL-2011-219-8-1 x Pusa Purple Long | BL-219 x S-324-465-2-2 |  |
|  | SL-8-PB-1-3-1-4 x Pusa Purple Long | Pusa Purple Round x S-324-465-2-2 |  |
| Anthocyanin content (%) | BL-2011-219-8-1 x Pusa Purple Long | SL-8-PB-1-3-1-4 x Pusa Purple Round |  |
|  | SL-8-PB-1-3-1-4 x Pusa Purple Long | SL-8-PB-1-3-1-4 x Pant Rituraj |  |
|  | Pant Rituraj x S-324-465-2-2 | Pusa Purple Long x BL-219 |  |
